# What is the evidence for interactions between filaggrin null mutations and environmental exposures in the aetiology of atopic dermatitis? A systematic review†

**DOI:** 10.1111/bjd.18778

**Published:** 2020-02-11

**Authors:** H. Blakeway, V. Van‐de-Velde, V.B. Allen, G. Kravvas, L. Palla, M.J. Page, C. Flohr, R.B. Weller, A.D. Irvine, T. McPherson, A. Roberts, H.C. Williams, N. Reynolds, S.J. Brown, L. Paternoster, S.M. Langan, (on behalf of UK TREND Eczema Network)

**Affiliations:** ^1^ Faculty of Health Sciences University of Bristol Bristol Medical School Oakfield House Oakfield Grove Bristol BS8 2BN U.K.; ^2^ Department of Dermatology Lauriston Building, Lauriston Place Edinburgh EH3 9HA U.K.; ^3^ Department of Infection St. Thomas’ Hospital Westminster Bridge Rd Lambeth, London SE1 7EH U.K.; ^4^ Department of Medical Statistics London School of Hygiene and Tropical Medicine London U.K.; ^5^ School of Public Health and Preventive Medicine Monash University Level 4, 553 St Kilda Road Melbourne 3004 Australia; ^6^ Unit for Population‐Based Dermatology Research St John's Institute of Dermatology Guy's & St Thomas’ NHS Foundation Trust & King's College London Strand, London WC2R 2LS U.K.; ^7^ Clinical Medicine Trinity College Dublin Dublin Ireland; ^8^ The National Children's Research Centre Crumlin Ireland; ^9^ Dermatology Children's Health Ireland Crumlin Ireland; ^10^ Churchill Hospital Old Road Headington, Oxford OX3 7LE U.K.; ^11^ Nottingham Support Group for Carers of Children with Eczema Nottingham U.K.; ^12^ Centre of Evidence‐Based Dermatology University of Nottingham Nottingham NG7 2NR U.K.; ^13^ Dermatology Royal Victoria Infirmary NHS Foundation Trust Newcastle upon Tyne U.K.; ^14^ Institute of Cellular Medicine Faculty of Medical Sciences Newcastle University Newcastle upon Tyne U.K.; ^15^ Skin Research Group, Division of Molecular and Clinical Medicine School of Medicine University of Dundee Dundee DD1 9SY U.K.; ^16^ Department of Dermatology Ninewells Hospital Dundee DD1 9SY U.K.; ^17^ MRC Integrative Epidemiology Unit at the University of Bristol Population Health Sciences Bristol Medical School, Oakfield House, Oakfield Grove Bristol BS8 2BN U.K.; ^18^ Faculty of Epidemiology and Population Health London School of Hygiene and Tropical Medicine London WC1E 7HT U.K.; ^19^ Health Data Research UK London U.K.

## Abstract

**Background:**

Epidemiological studies indicate that gene–environment interactions play a role in atopic dermatitis (AD).

**Objectives:**

To review the evidence for gene–environment interactions in AD aetiology, focusing on filaggrin (*FLG*) loss‐of‐function mutations.

**Methods:**

A systematic search from inception to September 2018 in Embase, MEDLINE and BIOSIS was performed. Search terms included all synonyms for AD and filaggrin/*FLG*; any genetic or epidemiological study design using any statistical methods were included. Quality assessment using criteria modified from guidance (ROBINS‐I and Human Genome Epidemiology Network) for nonrandomized and genetic studies was completed, including consideration of power. Heterogeneity of study design and analyses precluded the use of meta‐analysis.

**Results:**

Of 1817 papers identified, 12 studies fulfilled the inclusion criteria required and performed formal interaction testing. There was some evidence for *FLG*–environment interactions in six of the studies (*P*‐value for interaction ≤ 0·05), including early‐life cat ownership, older siblings, water hardness, phthalate exposure, higher urinary phthalate metabolite levels (which all increased AD risk additional to *FLG* null genotype) and prolonged breastfeeding (which decreased AD risk in the context of *FLG* null genotype). Major limitations of published studies were the low numbers of individuals (ranging from five to 94) with AD and *FLG* loss‐of‐function mutations and exposure to specific environmental factors, and variation in exposure definitions.

**Conclusions:**

Evidence on *FLG*–environment interactions in AD aetiology is limited. However, many of the studies lacked large enough sample sizes to assess these interactions fully. Further research is needed with larger sample sizes and clearly defined exposure assessment.

**Linked Comment:** Park and Seo. *Br J Dermatol* 2020; **183**:411.

## Abstract 2

**What's already known about this topic?**

- Gene–environment interactions are considered important in the aetiology of atopic dermatitis.
- Loss‐of-function mutations in the gene coding filaggrin (*FLG*) are the most consistently reported genetic variants for atopic dermatitis.
- Studies have reported evidence for gene–environment interaction involving *FLG* and a range of different environmental exposures.

**What does this study add?**

- There is some evidence for *FLG*–environment interactions in the aetiology of atopic dermatitis; however, the evidence is limited.
- Studies lack large enough sample sizes to achieve adequate power in order to assess these interactions fully.

**Linked Comment:** Park and Seo. *Br J Dermatol* 2020; **183**:411.

Plain language summary available online

Atopic dermatitis (AD), also known as eczema or atopic eczema, is a complex, multifactorial, often debilitating disease.^1^ The prevalence of AD has risen rapidly, suggesting that environmental factors might be responsible for such changes.^2^ It is estimated that up to 20% of children and 3% of adults in high‐income countries are affected by AD.^3^ In order to discover ways to reduce the personal and public health burden, it is necessary to gain a better understanding of the aetiology of AD.

Considerable phenotypic heterogeneity, evidence for multiple genetic risk mechanisms^4^ and incomplete penetrance have led to complexities in understanding the genetic basis of AD.^5^ There have been 31 risk loci identified for AD to date.^4^ Loss‐of‐function mutations in the gene encoding filaggrin (*FLG*) are the strongest and most significantly associated genetic variants for AD.^6^

Profilaggrin is an insoluble protein found in the outer epidermis; monomeric filaggrin has multiple functions including aggregation of keratin filaments.^7^ *FLG* is essential for normal epidermal barrier function and formation,^8^ contributing to the skin water‐holding capacity and pH balance.^9^ The two most prevalent loss‐of‐function mutations in *FLG* in white European populations are R510X and 2282del4, present in approximately 9% of healthy people in Northern European populations. These mutations are strongly associated with AD risk, particularly early‐onset and severe disease.^6^ Other, less prevalent, loss‐of‐function mutations in *FLG* have been identified.^10^, ^11^, ^12^ Despite increased understanding of the importance of genetic factors, the rising AD prevalence has been too substantial and rapid to be explained purely by genetic factors.^13^ Environmental factors have been implicated in the rising AD prevalence; exposure to such factors *in utero* or in later life may play a role in AD aetiology.^14^

Gene–environment interaction (GEI) may be defined as occurring when individuals with different genotypes respond to an environmental exposure in different ways; this interaction contributes to many common phenotypes and complex genetic traits. There is evidence that GEI plays a role in atopic diseases, such as asthma, in which genotype interacts with environmental factors, including maternal smoking and house dust mite.^15^, ^16^ A recent review on allergic diseases highlighted that several studies exploring GEI in AD exist, yet findings have not been synthesized (e.g. in a systematic review).^17^ Our objective was to perform a systematic review of the evidence for GEI in AD, focusing on interactions with the *FLG* null genotype, because this is the strongest and most widely replicated AD genetic risk factor, and because the role of filaggrin in skin barrier function provides a priori support for a hypothetical GEI effect.

## Materials and methods

This systematic review was prospectively registered (PROSPERO ID CRD42017057818). A detailed electronic search of MEDLINE and Embase via Ovid, and BIOSIS via Web of Science was undertaken from inception of each database to September 2018, identifying manuscripts in any language. To define AD, the search terms ‘atopic dermatitis’, ‘atopic eczema’ and ‘eczema’ were used, and to define *FLG* mutations, ‘filaggrin’, ‘*FLG*’, possible misspellings and previously reported *FLG* mutation names were included in the strategy (Methods S1; see Supporting Information). We focused on incident AD cases, including studies that examined interactions for AD development rather than interactions for established AD. To avoid defining an exhaustive list of environmental factors a priori, the inclusion of any environmental factor was assessed during the title and abstract screening. We defined environmental exposures as proposed by Rothman.^18^ The primary outcome measure was evidence of a statistically significant (defined as *P* < 0·05) *FLG–*environment interaction in the aetiology of AD and the secondary outcome was the strength of the association of the interaction (evidence of a dose–response relationship) and AD severity. Details of abstract screening, inclusion and exclusion criteria and data extraction are provided in Methods S2 and Table S1 (see Supporting Information).

Quality and bias assessment was performed using criteria modified from guidance for nonrandomized studies to determine quality of studies; ROBINS‐I to assess risk of bias in nonrandomized studies of interventions, and HuGENet for genetic studies, including assessing whether confounders were considered.^19^, ^20^ These two tools were combined to determine bias in genetic and environmental studies, as neither tool was designed for GEI studies.

Post hoc sample‐size calculations were undertaken to estimate the sample size required to detect a GEI effect varying between 1·2 and 2^6^ in a case–control/cohort study for a binary single‐nucleotide polymorphism (SNP) and binary exposure under a series of assumptions for model parameters (Table S2; see Supporting Information), using *R* 3·5·0 (*R* package powerGWASinteraction).

## Results

**Figure 1:**
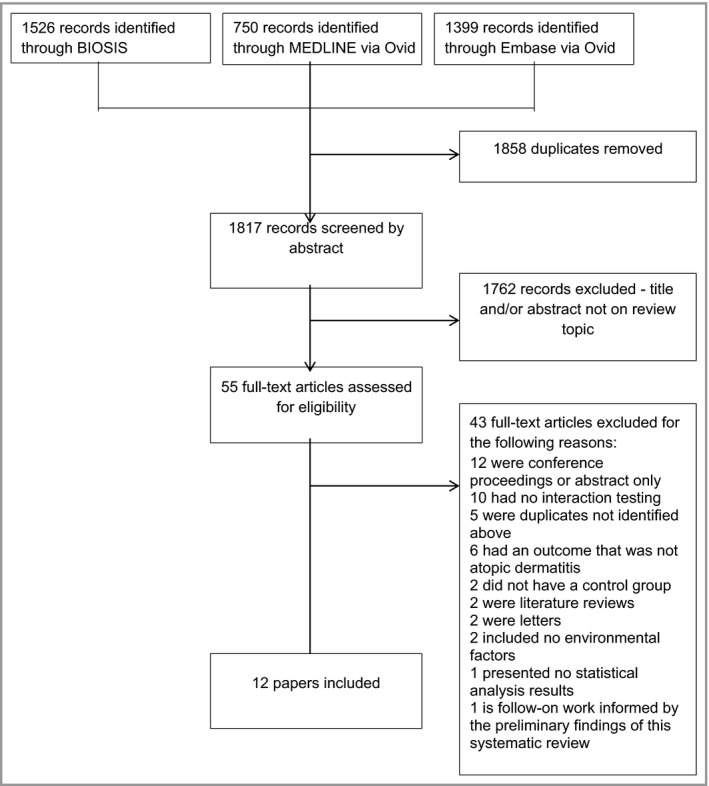
Flowchart showing systematic review.

The search identified 1817 papers of possible relevance (Fig. 1); 12 met our inclusion criteria (Table S1; see Supporting Information). Papers tested various environmental exposures (Table 1).

**Table 1:** Environmental exposures assessed in included studies

Environmental exposures assessed in one paper	Environmental exposures assessed in two or more papers
Older siblings and daycare attendance^a^,^27^ sex,^28^ maternal parity,^28^ maternal AD,^28^ maternal smoking,^28^ environmental tobacco smoke exposure in early life,^28^ birth year,^31^ serum vitamin D levels,^33^ maternal IgE sensitization^29^	Early‐life cat exposure^a^,^24^, ^25^ breastfeeding^a^,^28^, ^30^ phthalate exposure in urine metabolites and household dust^a^,^35^, ^36^ water hardness^a^,^26^, ^37^

a Environmental exposures where significant interactions with *FLG* were reported (*P* < 0·05).

The study designs of these 12 papers included 10 cohort studies, one case–control study and one family‐based study (Table S3; see Supporting Information). Study populations ranged from 296 to 5188 individuals and participant ages ranged from 1 month to 69 years. The number of participants in each study with *FLG* loss‐of‐function mutations ranged from 27 (9·1%) to 459 (10·2%). The AD definition and method of ascertainment varied between studies (Table S4; see Supporting Information).^21^, ^22^, ^23^ None of the included studies investigated the strength of interaction or AD severity.

Of the 12 publications, including 15 studies (Table S4; see Supporting Information), six studies showed evidence for GEI (*P* < 0·05) (Table 1). Most studies used regression models to calculate *P*‐values and some presented hazard ratios (HRs). Heterogeneity in study design and exposures precluded formal meta‐analysis.

### Cat exposure

Two studies assessed the *FLG*–cat interaction.^24^, ^25^ Bisgaard *et al*. tested for an interaction in the Copenhagen Prospective Study on Asthma in Childhood (COPSAC) (*n* = 379) and reported an increased risk related to cat exposure at birth among children aged 0–5 years who had *FLG* null mutation [HR 11·11, 95% confidence interval (CI) 3·79–32·60; *P*_interaction_ = 0·0008]; findings were replicated in the Manchester Asthma and Allergy Study (MAAS) (*n* = 503), with an increased risk owing to interaction of cat exposure at birth and *FLG* null genotype (HR 3·82, 95% CI 1·35–10·81; *P*_interaction_ = 0·011). Schuttelaar *et al*. (*n* = 934) reported no overall interaction (*P* = 0·85) between *FLG* null genotype (one/two *FLG* loss‐of‐function mutations) and cat exposure at home [odds ratio (OR) with *FLG* loss‐of‐function mutation(s) and cat exposure = 1·9; OR for *FLG* wild‐type individuals and cat exposure = 2·1]. However, Schuttelaar *et al*. reported an interaction when examining the 2282del4 mutation only (*P* = 0·003), with a stronger effect in children aged 0–8 years with a cat at home (OR 6·0, 95% CI 3·2–11·3) compared with those without (OR 2·2, 95% CI 1·4–3·7).^25^ All those with 2282del4 mutations were heterozygous.^25^ As all *FLG* loss‐of‐function mutations have biological equivalence on filaggrin protein expression, there is no clear biological plausibility for an interaction with one mutation and not another. Evidence for GEI comes from small numbers of individuals with *FLG* mutation, cat exposure and development of AD. In Bisgaard *et al*. five such individuals were reported. Schuttelaar *et al*. did not provide the number of individuals, but it can be inferred that *n* < 84 for the overall interaction and *n* < 50 for the 2282del4 interaction.^24^, ^25^

### Dog exposure

Bisgaard *et al*. tested for an interaction between *FLG* loss‐of‐function mutations and dog ownership in the first year of life. There was no evidence for an interaction in COPSAC (*n* = 379) (result statistics not reported) or MAAS (*n* = 503) (HR 0·59, 95% CI 0·16–2·20; *P* = 0·43).^24^

### Siblings

One study reported an interaction between *FLG* genotype and presence of older siblings among both children attending and those not attending daycare at 2 years of age in two separate studies – LISAplus cohort (aged 6–72 months, *n* = 1037, interaction OR 3·27, 95% CI 1·14–9·36; *P* < 0·05) and GINIplus cohort (12–72 months, *n* = 1828, interaction OR 2·41, 95% CI 1·06–5·48; *P* < 0·05).^26^ This interaction increased the risk of AD.^27^

### Parity, maternal atopy and child's sex

Henderson *et al*. found no evidence of an interaction between *FLG* genotype with parity (*n* = 4463, *P* = 0·802), maternal asthma or AD (*n* = 5188, *P* = 0·486 and *P* = 0·884, respectively) or the child's sex (*P* = 0·959)^28^ in children aged 6 months to 11 years who were part of a prospective cohort study.

### Maternal IgE sensitization

Esparza‐Gordillo *et al*. conducted a parent‐of‐origin analysis investigating the effect of a child's *FLG* genotype and maternal and paternal *FLG* genotypes on the child's AD risk (*n* = 1209 families).^29^ Although interactions were not the focus of this study, results were stratified by maternal IgE sensitization status, allowing us to compare the effect of the child's *FLG* genotype in those whose mothers were or were not IgE sensitized. The child's *FLG* genotype had a stronger risk effect when mothers had normal IgE levels [relative risk (RR) for one *FLG* loss‐of‐function mutation = 2·30 (95% CI 1·64–3·22); RR for two *FLG* loss‐of‐function mutations = 7·19 (95% CI 3·77–13·7)] compared with those who had sensitized mothers [RR1 1·37 (95% CI 0·97–1·94); RR2 2·98 (95% CI 1·19–7·45)]. However, CIs were wide and overlapping. Esparza‐Gordillo *et al*. reported the opposite for effect of maternal genotype, so it is unclear whether this evidence points to a true interaction with exposure to mothers with elevated IgE or whether the observation results from maternal genetic effects *in utero* or imprinting effects of *FLG* genotype.^29^

### Smoking

One study tested for possible interactions with *FLG* mutations and maternal smoking during pregnancy (*n* = 5140) or childhood environmental tobacco smoke exposure (*n* = 4874). Their results showed no evidence for either interaction (maternal smoking *P* = 0·362, child environmental tobacco smoke exposure *P* = 0·742).^23^

### Breastfeeding

Ziyab *et al*. found evidence for a protective association between breastfeeding duration and AD in children aged 1 or 2 years (*n* = 885) carrying at least one *FLG* loss‐of‐function mutation (*P* = 0·02), with no evidence in those without an *FLG* null mutation (*P* = 0·64).^30^ However, this was a stratified analysis and formal interaction testing was not undertaken. Henderson *et al*. found no evidence for this interaction (*P* = 0·952) in their earlier, larger study (*n* = 5158).^28^

### Birth year

Thyssen *et al*. investigated *FLG* and year of birth in adults aged 18–69 years (*n* = 3202) but did not report any evidence of interaction (*P* = 0·19) on AD risk.^31^

### Water hardness

Interaction of *FLG* genotype and water hardness has been investigated in two papers within the same cohort. Perkin *et al*. investigated the association between calcium and chlorine levels in water and AD during the first 3 months of life in the Enquiring About Tolerance study, a U.K. population‐based cohort of 1303 infants. Interaction tests did not show a statistically significant interaction between *FLG* genotype and high calcium‐low chlorine concentration in water, low calcium‐high chlorine or high calcium‐high chlorine concentrations.^26^ They subsequently reported evidence of an interaction between *FLG* loss‐of‐function mutations and water hardness increasing AD risk, when studying high calcium concentrations in water (> 256 mg L^−1^ CaCO_3_) from 3 months of age in this cohort (*N* = 1303; *n* = 75 with *FLG* null mutation exposed to high CaCO_3_ levels, *P* = 0·008).^32^

### Vitamin D

A possible interaction between serum vitamin D levels and *FLG* genotype was investigated by Berents *et al*. They measured serum vitamin D levels in 558 participants at age 1–13 months and 2 years alongside interviews assessing vitamin D intake. They did not find any evidence for an interaction (*P* > 0·13).^33^

### Urine phthalate metabolites and household dust phthalate

Phthalates are added to plastic to increase flexibility. They have been reported to be associated with childhood AD.^34^ Wang and Karmaus investigated whether there was an interaction between urine phthalate metabolite levels and *FLG* genotype in children aged 3 years in the aetiology of AD (*n* = 453). They studied four phthalates, monoethyl phthalate, monobutyl phthalate (MBP), monobenzyl pthathlate (MBzP) and mono(2‐ethyl‐5‐hydroxyhexyl) phthalate, which they classified as lower or higher levels in relation to the median. They reported evidence for an interaction between the P478S genotype TT and phthalates MBP (*P* = 0·015) and MBzP (*P* = 0·018)^35^ and increased AD risk; however, they did not replicate their findings or perform corrections for multiple testing.^35^ A similar interaction was investigated by Ait Bamai *et al*. who assessed seven phthalates found in household dust, and 11 phosphorus flame retardants. They found evidence for an interaction between *FLG* loss‐of‐functions and diisononyl phthalate (*P* = 0·039).^36^ The group also reported a nonsignificant negative dose–response relationship among children with *FLG* loss‐of‐function mutation(s) in a categorical model (first quartile compared with fourth quartile, *P* for trend = 0·087). This analysis was undertaken on a sample size of five children with AD and *FLG* null genotypes and the researchers did not correct for multiple testing.^36^

### Quality of studies

Most studies included unselected cases from the general population or cohorts, and controls were selected from the same population as those with AD. *FLG* genotype was assessed using accepted methodology (Table S3; see Supporting Information). The timing and method of assessment of environmental exposures was variable; studies may be vulnerable to reverse causality owing to exposure status being assessed after AD onset. Participants in each study were of homogeneous ethnicity and analyses were adjusted for age. Details of confounder adjustment were missing in five of 12 included studies (Table S3; see Supporting Information). Studies varied in their presentation of interaction results, with some providing only a *P*‐value or statement of statistical significance and others also providing effect estimates and CIs across strata and a *P*‐value for the interaction term or statement of statistical significance (Table S4; see Supporting Information). None of the included studies adjusted for multiple testing and only Berents *et al*. reported power calculations.^33^ The results of the studies supported their conclusions; however, they were underpowered and therefore the results of these studies must be interpreted with caution. Under reasonable assumptions on the magnitude of main effects, prevalence of AD, environmental exposure and SNP allele frequency, post hoc power calculations (Table S2; see Supporting Information) indicate that the sample size required to detect an interaction with OR ~2·0 is approximately 5000 individuals, whereas a sample size of approximately 63 000 individuals is required to detect a more modest GEI effect with OR 1·2.

## Discussion

Our results highlight important challenges when studying GEIs in the aetiology of AD. We identified only 12 articles that reported *FLG*–environment interactions in AD. Our initial search strategy returned many results, but the majority were excluded because they lacked information essential to the review. Evidence was found for interactions between *FLG* genotype and breastfeeding duration, older siblings, phthalate exposure in household dust and urine phthalate metabolite levels, early‐life exposure to cats, and water hardness. All interactions increased the risk of AD apart from prolonged breastfeeding, which decreased the risk. Owing to very limited evidence in support of these interactions, small numbers and lack of replication (one study undertook replication) it is difficult to interpret the results, and findings must be interpreted with caution. Table 2 shows our suggestions of the components required for a rigorous GEI study, which may improve conclusions in future studies.

**Table 2:** Components required for rigorous gene–environment interaction (GEI) study

Suggestion	Reason
Large sample size with mutation and environmental exposure (Table S2; see Supporting Information)	Sample size must be large enough to detect true gene by environment interaction effect
Design the study basing the sample size on power to detect interaction effect	Design the study and basing the sample size on power to detect a main effect will likely result in insufficient sample size
Use accepted diagnostic criteria for atopic dermatitis	This reduces the possibility of introducing heterogeneity into the results
Use robust methods of exposure measurement	Questionnaires or indirect measurements of exposure can introduce variation and recall bias into the results. Using validated tools will help reproducibility and reduce information bias
Collect measurements of exposure at defined time periods across the study population	This avoids variation in the timing of exposure measurements influencing disease risk
Correct for multiple testing and publication bias	This reduces the possibility of interpreting chance results as positive findings
Tailor studies to different ethnic groups currently not covered by research	Increasing diversity in genetic research will enable us to understand the importance of GEIs in populations of different ethnicities

Our review has several strengths. A detailed search strategy was used to identify all relevant papers. Screening and data extraction were carried out in duplicate, with secondary resolution of conflicts, reducing the possibility of introducing bias by systematically selecting certain papers. The majority (10 of 12, 83%) of studies used data from cohort studies, thus they were able to consider temporality, and researchers mostly measured the outcome ‘AD’ using validated criteria.

Our findings should be considered in light of some limitations. Many studies were excluded from the review as they did not specifically test for GEI in their analyses. Studies were also excluded if they measured indirect outcomes of AD by examining IgE levels, transepidermal water loss, or skin‐prick tests, which are not measures of the outcome (AD), but responses to exposures. Many of the included studies performed GEI analysis as a secondary analysis, e.g. Berents *et al*.,^33^ meaning they did not aim to have sufficient power to assess GEIs; hence, the importance of measuring GEIs as primary outcome (Table 2). We were unable to evaluate the risk of reporting biases formally, so we cannot rule out the possibility that studies which found nonsignificant interactions yet failed to report such results are missing from our review. Many of the studies included in this review relied on population data and there may be heterogeneity in outcome definition (Table 2).^38^ The predefined scope of this review was to investigate *FLG–*environment interactions, which by definition excluded the study of effects within populations where *FLG* null mutations are not prevalent or have not been identified.^7^, ^39^ Many large genetic studies have been conducted within populations of European ancestry, where *FLG* null mutations are prevalent. Ongoing work to increase diversity in genetic research^40^ will allow future investigations of GEI in populations of all ethnicities (Table 2).

Heterogeneity in methodology between published studies and the limited number of studies assessing the same exposure precluded meta‐analysis or formal assessment of publication bias. None of the included studies reported correcting for multiple testing; hence, interaction effects could be a result of chance. It is unclear how many studies had predefined hypotheses, which risks introducing reporting bias. Replication of findings was limited and in some cases where two studies investigated the same interaction, discordant findings were seen, such as Ziyab *et al*. (*FLG* genotype and breastfeeding) and Henderson *et al*.^28^, ^30^

One reason for the limited evidence for GEI and lack of replication is lack of statistical power. Detailed review showed that the number of individuals on which the interaction analysis was based (i.e. cases with both exposure and *FLG* null genotype) was small; hence, included studies were likely to be underpowered. The number of individuals with both a *FLG* loss‐of‐function mutation and exposure to the specific environmental factor was not always specified, but in those studies that did specify this number, it ranged from *N* = 5 to *N* = 167.^24^, ^28^

In complex diseases such as AD, where the main genetic effect sizes are small, a large sample size is necessary to detect small interaction effects.^41^ Researchers need to utilize sufficiently large sample sizes to detect GEIs, and generally investigators should demonstrate that their sample has adequate power to detect an interaction effect.^42^ Even in cases where meta‐analysis across studies is possible, results are not always meaningful owing to variable measurement of environmental exposures.^41^

Studies of GEI face inherent challenges in attempting to gain a full understanding of interactions because of the difficulty in uniformly measuring the environmental parameter, which in turn limits the understanding of the underlying disease mechanism.^43^ The difficulty in measuring exposure in GEI studies in AD is shown in the Wang *et al*. study, which tested for an interaction between phthalate exposure and *FLG* genotype. To measure exposure, phthalate metabolite levels were measured using urine samples.^36^ This is not a direct measure of the exposure; therefore, we questioned whether it should be included in the review. It provides only a moderate prediction of exposure owing to the short half‐life and rapid excretion of phthalates leading to considerable day‐to‐day variation.^36^ Other studies used different methods, such as questionnaires, to derive environmental exposure data retrospectively; this could introduce recall bias.^27^

Variation in the timing of environmental exposure is important in terms of influencing subsequent disease risk, as timing of exposure may not be accurately measured with methods such as infrequent questionnaires.^44^ Using robust validated measures of exposure reduces variation and aids reproducibility of results (Table 2). For some of these exposures it is easy to hypothesize a biological explanation as to why people with *FLG* haploinsufficiency might have different responses; for example, pet exposure and older siblings could act via microbial exposure, as proposed by the hygiene hypothesis.^45^ With other possible interactions such as urine phthalate metabolites, it is harder to hypothesize plausible mechanisms. Cohort studies may be vulnerable to reverse causality when assessing early‐life exposures, as, although outcomes were measured after the exposure in the majority of studies, there remains a possibility that early signs of AD, or the presence of older siblings with AD, influenced the behaviour of parents who subsequently modified the exposure.

GEIs are widely viewed as important in the aetiology of AD. However, the limited evidence and lack of power of published studies to detect GEI effects, as indicated by the sample‐size calculations we carried out, highlights the importance of further research. Such research is needed to test for replication of interactions reported to date (Table 1) using larger sample sizes. Furthermore, unexplored GEIs may also warrant investigation, including genetic risk variants in addition to *FLG* loss‐of‐function mutations. The Early Genetics and Lifecourse Epidemiology consortium is investigating possible GEIs with selected SNPs associated with AD. Our recommendations for future studies of GEIs can be shown in Table 2, which would improve the quality of evidence and enable us to draw more robust conclusions about the nature of GEIs. Together this work will improve understanding of GEI in the aetiology of AD, which will help to inform both public health and individual lifestyle decisions.

## Supporting information


**Methods S1** Search strategies.
**Methods S2** Method of screening and data extraction of relevant studies.
**Table S1** Inclusion and exclusion criteria.
**Table S2** Sample size required to detect gene–environment interaction.^46^, ^47^
**Table S3** Characteristics of studies of loss‐of‐function mutations in *FLG* and gene‐environment interaction in atopic dermatitis.
**Table S4** Studies examining the interactions between *FLG* mutations and environmental exposure and risk of atopic dermatitis.

**Powerpoint S1** Journal Club Slide Set.

## Funding sources

M.J.P. is supported by an Australian National Health and Medical Research Council Early Career Fellowship (1088535). C.F. is funded through a U.K. National Institute for Health Research (NIHR) Career Development Fellowship (CDF‐2014‐07‐037) and is also supported by the NIHR Biomedical Research Centre based at Guy's and St Thomas’ NHS Foundation Trust and King's College London. S.J.B. holds a Wellcome Trust Senior Research Fellowship in Clinical Science (106865/Z/15/Z) and reports research funding from the British Skin Foundation, Tayside Dermatology Research Charity and a researcher‐initiated grant from Pfizer. L. Paternoster is supported by an Academy of Medical Sciences Springboard Award, which is supported by the Wellcome Trust, The Government Department for Business, Energy and Industrial Strategy, the Global Challenges Research Fund and the British Heart Foundation (SBF003\1094). L. Paternoster works in the MRC Integrative Epidemiology Unit, which receives funding from the U.K. Medical Research Council (MC_UU_00011/1). S.M.L. reports grants from the Wellcome Senior Clinical Fellowship in Science (205039/Z/16/Z) during the study period. N.R. is a NIHR Senior Investigator and is also supported by the Newcastle NIHR Biomedical Research Centre, the Newcastle MRC/EPSRC Molecular Pathology Node and the Newcastle NIHR Medtech and *In vitro* diagnostic Co‐operative. S.J.B., L. Paternoster and S.M.L. are investigators for the European Union Horizon 2020‐funded BIOMAP Consortium (http://www.biomap-imi.eu/). The Wellcome Trust, BIOMAP, NIHR and the British Association of Dermatologists played no role in the development or results of this study, and all authors carried out this research independently of the funding bodies. The findings and conclusions in this report are those of the authors and do not necessarily represent the views of the funders.

## Authors’ contributions

H.B. contributed to the design of the study, was the guarantor, developed the search strategy and the PROSPERO protocol, drafted the published protocol, led data extraction and critical appraisal and drafted the final manuscript. V.V., V.B.A. and G.K. contributed to the abstract screening, data extraction and critical appraisal and critically reviewed the manuscript. M.J.P. developed the search strategy and critically reviewed the manuscript. L. Palla contributed to the design of the study, interpretation of analysed data and statistical calculations, and critically reviewed the manuscript. C.F. contributed to the design of the study and critically reviewed the manuscript. R.B.W. contributed to the design of the study and critically reviewed the manuscript. A.D.I. refereed the data extraction and critically reviewed the manuscript. T.M., A.R., H.C.W. and N.R. critically reviewed the manuscript. S.J.B., L. Paternoster and S.M.L. contributed to the design of the study, developed the search strategy and the PROSPERO protocol, performed, supervised and refereed data extraction, reviewed data analysis, contributed to drafting the manuscript and critically reviewed the final manuscript. All authors approved the final manuscript as submitted.

## Data access, responsibility and analysis

H.B. (Faculty of Health Sciences, University of Bristol, Bristol Medical School, Oakfield House, Oakfield Grove, Bristol, BS8 2BN, U.K.) and S.M.L. (Department of Non‐Communicable Disease Epidemiology, London School of Hygiene and Tropical Medicine, London, WC1E 7HT, U.K.) had full access to all the data in the study and take responsibility for the integrity of the data and the accuracy of the data analysis. The corresponding author and guarantor (H.B.) affirms that this manuscript is an honest, accurate and transparent account of the study being reported; that no important aspects of the study have been omitted and that any discrepancies from the study as planned (and, if relevant, registered) have been explained.

## Data sharing

Our OVID MEDLINE search strategy has been registered on PROSPERO (https://www.crd.york.ac.uk/prospero/) and all data are publicly available.
