# Supplementary material for: What is the evidence for interactions between filaggrin null mutations and environmental exposures in the aetiology of atopic dermatitis? A systematic review
Source: Br J Dermatol. 2020 Feb 11;183(3):443–51. doi: 10.1111/bjd.18778 (PMC7496176; doi:10.1111/bjd.18778)
Supplement: Supplementary file 1 — Methods S1 Search strategies. Methods S2 Method of screening and data extraction of relevant studies. Table S1 Inclusion and exclusion criteria. Table S2 Sample size required to detect gene–environment interaction.46, 47 Table S3 Characteristics of studies of loss‐of‐function mutations in FLG and gene‐environment interaction in atopic dermatitis. Table S4 Studies examining the interactions between FLG mutations and environmental exposure and risk of atopic dermatitis. [file BJD-183-443-s001.docx]

*Supplementary methods 1: Search strategies*

**MEDLINE (via Ovid) search strategy**

1. Eczema/
2. eczema.ab,kf,ti.
3. Dermatitis, Atopic/
4. dermatitis, atopic.ab,kf,ti.
5. atopic dermatitis.ab,kf,ti
6. atopic eczema.ab,kf,ti
7. filaggrin.ab,kf,ti
8. FLG.ab,kf,ti
9. Filagrin. ab,kf,ti
10. Fillagrin. ab,kf,ti
11. Fillaggrin. ab,kf,ti
12. 1 OR 2 OR 3 OR 4 OR 5 OR 6
13. 7 OR 8 OR 9 OR 10 OR 11
14. 12 AND 13
15. Remove duplicates from 14

**EMBASE (via Ovid) search strategy**

1. Eczema/
2. eczema. ab,kw,ti
3. Dermatitis, Atopic/
4. dermatitis, atopic.ab,kw,ti
5. atopic dermatitis.ab,kw,ti
6. atopic eczema. ab,kw,ti
7. filaggrin. ab,kw,ti
8. FLG. ab,kw,ti
9. Filagrin. ab,kw,ti
10. Fillagrin. ab,kw,ti
11. Fillaggrin. ab,kw,ti
12. 1 OR 2 OR 3 OR 4 OR 5 OR 6
13. 7 OR 8 OR 9 OR 10 OR 11 OR 12
14. 12 AND 13
15. Remove duplicates from 14

**BIOSIS Citation Index search strategy**

1. TS=((eczema OR “atopic dermatitis” OR “atopic eczema” OR dermatitis))
2. TS=((filaggrin OR FLG OR filagrin OR fillagrin OR fillaggrin))

(#1) AND (#2)

*Supplementary Material Methods 2: Method of screening and data extraction of relevant studies*

Authors V.A, H.B and G.K and V.V in groups of two and later SJB, LaP and SML independently screened all abstracts and any potentially relevant full text articles using the online software Covidence (https://www.covidence.org). Disagreements were resolved through discussion with the wider group to gain consensus. Removal of exact duplicates took place using authors, title, journal, volume, journal issue and page numbers. Inclusion and exclusion criteria are detailed in supplementary Table 1. Studies were excluded if they did not include a formal assessment of *FLG*-environment interaction, at a minimum reporting results in one of three formats: (1) a p value for an interaction term^46^, (2) genetic association results stratified according to environmental exposure or (3) environmental association results stratified by genotype. In cases where studies reported having tested for GEI but did not include results, authors were contacted for further information, and studies providing that information were included. To maximise inclusion of all relevant studies, the systematic review team searched review papers (which were to be excluded) for references to additional studies missed by the search strategy.

Data from included papers were extracted independently by the same two groups of researchers using standardized extraction sheets. We determined the frequency of (i) different study designs, (ii) exposures with which the GEI was measured, (iii) outcomes (binary and categorical) and (iv) the statistical methods by which the interaction was assessed. We extracted data on effect estimates (odds ratios or risk ratios) observed for specific genetic variants and identified the level of reporting in the study as recommended by Knol *et al.^46^* We considered whether formal meta-analysis was appropriate by assessing whether multiple studies included the same scientific question and results were presented in a sufficiently consistent method to allow this.

*Supplementary Table 1: Inclusion and Exclusion Criteria*

| **Inclusion criteria** | **Exclusion criteria** |
| --- | --- |
| **Participants** |  |
| Studies involving participants of any age, ethnicity, or gender whose genetic data were included in the eligible studies. | No control group of participants. |
| **Exposure** |  |
| Any environmental factor, as proposed by Rothman, “Factors that are exogenous to and nonessential for the normal functioning of human beings and that alter patterns of disease and health”. |  |
| **Gene variants** |  |
| *FLG* sequence variants, including mutations of known functional significance (e.g. truncating mutations in exon 3) and sequence variants with no evidence of functional significance. |  |
| **Outcome** |  |
| Outcome of self-reported or clinician-diagnosed AD and/or severity. | Outcome not atopic AD, e.g. studies of localised AD such as contact dermatitis. |
| **Methodology used** |  |
| All types of methodological approaches to genetic studies in any language; including linkage studies, candidate gene association studies and genome-wide association studies. | No human data from human subjects presented, for example research which involved animal testing, *in vitro* testing or human simulation studies. |
| Study designs with interaction result report. | No interaction tested, or studies tested for an interaction but did not report the result and relevant data were not provided by authors. |
|  | Review papers, conference proceedings, books, letters or case reports. |

*Supplementary Table 2: Sample size required to detect GEIs with power=0.8, significance level=0.05 using 1-step approach (no screening) for a case-control design (or cohort design) candidate gene approach, assuming an allele frequency of 0.1*^47^*, a binary environmental exposure with prevalence=0.2, a prevalence of AD=0.2, an odds ratio of 1 (no association) between SNP and exposure, an odds ratio of 3.1*^6^ *for the genetic effect, an odds ratio of 1.1 for the environmental exposure and a case fraction in the sample of 0.2 (to reflect the prevalence as in a cohort study) and assuming only one candidate gene is being tested. The function powerGE in the R package ‘powerGWASinteraction’ was used*

| GEI effect | **Total Sample size required** |
| --- | --- |
| 1.1 | 227222 |
| 1.2 | 62937 |
| 1.3 | 30835 |
| 1.4 | 19026 |
| 1.5 | 13308 |
| 1.6 | 10067 |
| 1.7 | 8018 |
| 1.8 | 6640 |
| 1.9 | 5662 |
| 2 | 4936 |

*Supplementary Table 3: Characteristics of studies of loss-of-function mutations in FLG and gene-environment interaction in AD*

| Author, year, population source | Study design, Setting | Age at assessment of outcome | Ascertainment of environmental exposures- timing and their definition | Filaggrin mutation(s) | HWE described | Definition and ascertainment of AD | | | Method by which for GEI assessed | Confounders adjusted for in analyses |
| --- | --- | --- | --- | --- | --- | --- | --- | --- | --- | --- |
| Pet exposures – CAT and DOG | | | | | | | | | | |
| Bisgaard, 2008 , Denmark and UK | Prospective cohort study – COPSAC and MAAS | 0-5 years | COPSAC- **Pet exposure**-binary- cat or dog living in the house at birth determined at the interview at the 1 month visit  Dichotomised as high and low levels above and below 75% quartile.  MAAS- **Pet exposures**- binary- cat or dog present in house at birth determined by questionnaire at 4wks after birth. | R501X  2282del4 (COPSAC also genotyped for R2448X and S3247X)  Mutations analysed together in a two level dominant genetic model | Not Listed | COPSAC – physician diagnosed AD based on Hanifin and Rajka criteria  MAAS -International Study on Asthma and Allergies in childhood (ISAAC) questionnaire of parentally reported AD, collected by age 1y | | | Multiple Cox Regression and Kaplan-Meier curves | Not listed |
| Schuttelaar, 2009, Netherlands | Prospective cohort study | 0-8 years | **Cat ownership** – binary variable of whether a cat present at home at three months of age | R501X  2282del4  R2447X  Mutations analysed together in a dominant model other than 2282del4 which was analysed separately | All SNPs were in HWE | UK Working Party’s Diagnostic Criteria for AD at 4 years  ISAAC questionnaire at 1 year and 8 years | | | Odds ratio and confidence interval | Not stated |
| Older siblings and day-care | | | | | | | | | | |
| Cramer, 2010, Germany | Prospective cohort study (LISAplus and GINIplus) | LISAplus 6-72 months  GINIplus 12-72 months) | **Number of elder siblings** at birth of the index child was reported and transferred into the dichotomised variable “elder siblings”. | R501X and 2282del4  Mutations analysed together. Children with either (or both) variants were classified as having the *FLG* loss-of-function mutation. Children having negative results for both variants or for 1 variant and the other missing were classified as individuals without *FLG* mutations. | Not Listed | Parental report of doctor-diagnosed AD and AD symptoms or Medical examination by a trained pediatrician according to the International Study of Asthma and Allergies in Childhood, phase II protocol | | | logistic regression analyse, generalized estimating equations (GEEs) autoregressive covariates matrix | Parental allergy sex, education of, maternal smoking during pregnancy, contact with dog or cat in the first year of life, maternal age at childbirth >30 years, day care attendance during the first 2 years of life, and study region.  GINIplus data different intervention formulas were considered as covariates. And analysis of the interaction ‘‘*FLG* mutations x cat contact in the first year of life,’’ was performed |
| Parity, sex, maternal atopy, tobacco exposure and breastfeeding | | | | | | | | | | |
| Henderson, 2008 , UK and Ireland | Prospective cohort study | 6 months to 11 years | **Maternal AD, sex, maternal smoking in pregnancy, child’s environmental tobacco exposure, parity, breastfeeding**. Method of exposure determination and timing not stated. Variables are binary | R501X and 2282del4  Mutations analysed together as AA Aa aa | No significant deviation from HWE | Defined individuals with AD as those with reports of flexural dermatitis at 2 time points between 6 and 42 months according to ISAAC protocol. Further AD noted at annual research clinics from 7 to 11 years | | | Multinomial logistic regression and chi squared | sex, maternal smoking in pregnancy, the child’s environmental tobacco exposure, maternal social class, parity, breast-feeding, central heating, and maternal age |
|  |  |  |  |  |  | |  |  |  |  |
| Ziyab, 2016, UK | Prospective cohort, UK | 1 or 2 years | **Breastfeeding,** continuous | R501X, 2282del, and S3247X  Children carrying the minor allele for at least one of the *FLG* variants were classified as having *FLG* haploinsufficiency. Mutations analysed together | Not listed | AD yes/no  Chronic relapsing, itchy dermatitis lasting more than 6 weeks with characteristic morphology and distribution | | | Poisson regression with robust error variance | Not listed |
| Birth year |  |  |  |  |  | |  |  |  |  |
| Thyssen, 2012 , Denmark | Cross-sectional cohort | 18-69 years | **Birth year** 1936-1949, 1950-1962, 1963-1975 and 1976-1988 | R501X and 2282del4  Mutations analysed together, all viewed as the same | Not listed | AD yes/no according to UK Working Party’s diagnostic criteria | | | Chi square test  Logistic regression | Not listed |
| Water Hardness | | | | | | | | | | |
| Perkin , 2016, UK | Cross-sectional cohort | 3 months old | **Calcium and Chlorine levels in the water** and AD effect in infancy  A baseline group of low CaCO3/low total chlorine (CaL/CIL) and high CaCO3/low total chlorine (CaH/ClL), low CaCO3/high total chlorine (CaL/CIH), and high CaCO3/high total chlorine (CAH/ClH) groups | R501X, 2282del4, R2447X, S3247X; ABI7900 HT  Mutations analysed together | Not listed | Defined as visible AD at enrolment of study – binary | | | Interaction term included | Sex, ethnicity, home location, maternal age, socioeconomic status. (Maternal age at leaving full-time education), ownership of a water softener, family history of AD and other allergic diseases, frequency of bathing, and use of topical moisturizers and bathing products |
| Jabbar-Lopez, 2019 , UK | Prospective cohort | 3-36 months | **Water hardness** dichotomised based on median reported values of calcium carbonate levels by home postcode | R501X, 2282del4, R2447X, 3673delC and 3702delG  Mutations analysed together in a dominant model | R501X: P=0.595  2282del4: P=0.548  R2447X: P=0.919  3673delC: not calculable  3702delG: not calculable | Combined outcome of parent-reported, doctor diagnosed AD or visible AD | | | Stratification for K-M plots  Interaction term for Cox model | Urban location, socioeconomic status and ethnicity |
| Vitamin D, maternal IgE, phthalate metabolites and phthalates in household dust | | | | | | | | | | |
| Berents, 2016, Norway | Cross-sectional and prospective cohort | Visit one 5.1 years  Visit two 24.7 years | **Vitamin D** measured from blood taken at 1-13 months and 2 years alongside structured interviews of the caregivers on Vitamin D intake. Categorised into high, medium and low Vitamin D levels | R501X, 2282del4, R2447X, S3247X  Mutations analysed together (not specifically stated). All participants were heterozygotes – dominant model used | Not listed | Hanifin and Rajka except 23 children with a history of physician diagnosed AE at first visit | | | Stratification by *FLG* status | Age, season of birth, sex, BMI, skin pigmentation type, parental atopy, parental income, season of examination and recruitment source |
| Esparza-Gordillo, 2015, Central and Northern European | Two family based cohort studies | Not listed | In utero immune environment as measured by **allergen-specific IgE** | 2282del4, R501X, R2447X and S3247X  Mutations analysed as a combined null genotype | Not listed | Dr diagnosed AD according to the UK working party criteria | | | Interaction term included in the analysis | Not listed |
| Wang, 2015, Taiwan | Nested case-control | 3 years | **Urine phthalate metabolite levels levels** (MEP, MBP, MBzP, and 5OHMEHP) were measured by  UPLC–MS/MS and split into categories | RS11584340 (P478S)  Mutations analysed together, TT, CC and TC viewed separately | Not Listed | AD yes/no according to Hanifin and Rajka criteria at age 3 years | | | Logistic regression | Gender, premature birth, maternal age and education, maternal history of atopy, family income, duration of breast feeding, number of older siblings, pet raising, environmental tobacco exposure (ETS), usage of carpets at home, and fungi on house walls |
| Bamai, 2018, Japan | Cross sectional cohort | 7 years | 7 phthalates and 11 phosphorus flame retardants in household dust determined by analysis of house dust samples | 3321delA, Q1701X, S2554X, S2889X, S3296X, and K4022X analysed together in a dominant model | Not listed | Self-administered Japanese version of ISAAC core questionnaire given to mothers of 7 year old children | | | Interaction term included in analysis | Sex, household income, maternal smoking, parental history of atopy |

*Supplementary Table 4: Studies examining the interactions between FLG mutations and environmental exposure and risk of AD*

| Exposure | *FLG* loss-of-function mutation | Study (lead author, year) | Sample size total (cases/controls) | Cases with environmental exposure + *FLG*^-^/total with *FLG*^-^mutations | Evidence of G*E interaction (p value, stratified results) | Findings including strength of interaction |
| --- | --- | --- | --- | --- | --- | --- |
| CAT | R501X, 2282del4, R2448X, S3247X | COPSAC (Bisgaard, 2008)^22^ | N=379 (105/274) | 5/40 | **P_INT_ = 0.0008**  *FLG*^-^ plus cat HR_INT_=11.11 (95% CI: 3.79-32.60) P<0.0001  Cat HR in *FLG*^-^ individuals = 7.49 (95% CI: 2.37-23.67) P=0.0006 | Report evidence for an association but interaction analysis based on N=5 exposed cases with *FLG* loss-of-function mutation |
|  | R501X, 2282del4 | MAAS  (Bisgaard, 2008)^22^ | N=503 (187/316) | 11/50 | **P_INT_= 0.011**  HR_INT_=3.82 (95% CI: 1.35-10.81)  Cat HR in *FLG*^-^ individuals = 2.47 (95% CI: 1.09–5.62), p = 0.03) | Report evidence for an association but interaction analysis based on 11 *FLG*^-^ cases who were exposed to cat. |
|  | R501X, 2282del4 R2447X | PIAMA  (Schuttelaar, 2009)^23^ | N=934 (175/759) | ?/84 | **P_INT_ (all 3 mutations)= 0.85**  *FLG*^-^ OR in cat exposed = 1.9 (95% CI: 1.6– 2.9) P = 0.0029  *FLG*^-^ OR in cat unexposed = 2.1 (95% CI: 1.1–3.8) P = 0.024  **P_INT_ (2282del4 only) = 0.003**  Children with a cat OR = 6.0 (95% CI: 3.2–11.3) P < 0.001  Children without a cat OR = 2.2 (95% CI: 1.4–3.7) P = 0.001 | Report evidence that cat exposure enhances the effect of *FLG* loss-of-function mutation(s) but only for 2282del4. |
| DOG | R501X, 2282del4, R2448X, S3247X | COPSAC  (Bisgaard, 2008)^22^ | N=379 (105/274) | 8/40 | No evidence for interaction. P value not reported. | No evidence for interaction, based on only 8 individuals with *FLG* loss-of-function mutations and exposure to dog |
|  | R501X, 2282del4 R2447X | MAAS  (Bisgaard, 2008)^22^ | N=503 (187/316) | 8/50 | **P_INT_ = 0.43**  HR_INT_ 0.59 (95% CI: 0.16-2.20) |  |
| OLDER SIBLINGS | R501X  2282del4 | LISAplus  (Cramer, 2010)^30^ | N=1039*  (?/?)  *Note 1037 included in analyses | ?/70 | **P_INT_ >0.05**  *FLG^-^* x siblings OR=1.51 (95% CI: 0.62-3.68)  **P_INT_ no day-care <0.05**  *FLG^-^* x siblings OR=3.27 (95% CI: 1.14-9.36) | Report evidence of a strong, interaction between the presence of elder siblings and the most common *FLG* loss-of-function mutations (R501X and 2282del4) on the development of AD until the age of 6 years particularly in those who do not attend day-care. |
|  | R501X  2282del4 | GINIplus  (Cramer 2010)^30^ | N=1828 (?/?)  *Note 1722 included in analyses | ?/109 | **P_INT_ <0.05**  *FLG^-^* x siblings OR= 2.38 (95% CI: 1.09-5.17)  **P_INT_ no day-care <0.05**  *FLG^-^* x siblings OR=2.41 (95% CI: 1.06-5.48) |  |
|  | R501X  2282del4 | LISAplus + GINIplus combined  (Cramer 2010)^30^ |  |  | **P_INT_ <0.05**  *FLG^-^* x siblings OR= 1.94 (95% CI: 1.09-3.45)  **P_INT_ no day-care <0.01**  *FLG^-^* x siblings OR= 2.69 (95% CI: 1.41-5.15) |  |
| PARITY | R501X 2282del4 | ALSPAC  (Henderson, 2008)^24^ | N=4463 (1445/3018) | 131/459 | **P_INT_ = 0.802** | Report no evidence for an interaction between parity and *FLG* loss-of-function mutations |
| SEX | R501X 2282del4 | ALSPAC  (Henderson, 2008)^24^ | N=5255 (1445/3810) | 99/459  (where exp=female sex) | **P_INT_ = 0.959** | Report no evidence for an interaction between sex and *FLG* loss-of-function mutations |
| MATERNAL ATOPY-ASTHMA  MATERNAL AD | R501X 2282del4 | ALSPAC  (Henderson, 2008)^24^ | N=5188 (1417/3771) | 25/456 | **P_INT_ = 0.486** | Report no evidence for an interaction between maternal asthma or maternal AD and *FLG* loss-of-function mutations |
|  | R501X 2282del4 | ALSPAC  (Henderson, 2008)^24^ | N=5188 (1430/3758) | 78/456 | **P_INT_ = 0.884** |  |
| MATERNAL IGE SENSITISATION |  | Central and Northern European families  (Esparza-Gordillo, 2010)^33^ | N=1209 families (?/?) | ?/? | In individuals with a sensitised mother:  RR1=1.37(95% CI: 0.97-1.94)  RR2=2.98 (95% CI: 1.19-7.45)  In individuals with an unsensitised mother:  RR1=2.30 (95% CI: 1.64-3.22)  RR2=7.19(95% CI: 3.77-13.7) | Report a stronger effect of *FLG* null genotype in children whose mothers are IgE unsensitised, but might be due to maternal or imprinting genetic effects. |
| TOBACCO EXPOSURE-  In utero  In early life | R501X 2282del4 | ALSPAC  (Henderson, 2008)^24^ | N=5140 (1414/3726)  *Note 717 included in analysis | 28/452 | **P_INT_ = 0.362** | Report no evidence for an interaction between maternal smoking during pregnancy and *FLG* loss-of-function mutations |
|  | R501X 2282del4 | ALSPAC  (Henderson, 2008)^24^ | N=4874 (1348/3526)  *Note 1035 included in analysis | 41/427 | **P_INT_ = 0.742** | Report no evidence for an interaction between child environmental tobacco smoke exposure and *FLG* loss-of-function mutations |
| BREASTFEEDING | R501X 2282del4 | ALSPAC  (Henderson, 2008)^24^ | N=5158  (1408/3750) | 167/448 | **P_INT_ = 0.952** | Report no evidence for an interaction between breastfeeding and *FLG* loss-of-function mutations |
|  | R501X  2282del, S3247X | Isle of Wight Birth Cohort  (Ziyab, 2016)^25^ | N=885 (?/?) | ?/94 | **P_INT_ = 0.02**  Breastfeeding (*FLG*^-^) P=0.02  Breastfeeding (*FLG* wild type) P=0.64 | Report that *FLG* variants modified the association between breastfeeding duration and AD |
| BIRTH YEAR | R501X 2282del4 | 2006-2008 cross- sectional study of Copenhagen  (Thyssen, 2012)^31^ | N=3202 (324/2878) | 56/257 | **P_INT_ = 0.19** | Report no evidence for an interaction between birth year and *FLG* loss-of-function mutations |
| WATER HARDNESS | R501X  2282del4 R2447X  S3247X | Enquiring about tolerance cohort, UK (EAT) high calcium low chlorine water  (Perkin, 2016)^29^ | N=1302 (317/985) | 34/141 | **P_INT_ = 0.17**  OR_INT_=2.10 (95% CI: 0.74-5.99) | Report that effect estimates for domestic water content and chlorine on visible AD were greater in children carrying *FLG* loss-of-function mutations, but formal interaction tests did not show any evidence for an interaction |
|  | R501X  2282del4 R2447X  S3247X | Enquiring about tolerance cohort, UK (EAT) low calcium high chlorine water  (Perkin, 2016)^29^ | N=1303 (317/986) | 24/141 | **P_INT_ = 0.75**  OR_INT_=0.83 (95% CI: 0.27-2.60) |  |
|  | R501X  2282del4 R2447X  S3247X | Enquiring about tolerance cohort, UK (EAT) high calcium high chlorine water  (Perkin, 2016)^29^ | N=1303 (317/986) | 40/141 | **P_INT_ = 0.59**  OR_INT_ = 1.32 (95% CI: 0.49-3.55) |  |
|  | R501X  2282del4 R2447X  S3247X | Enquiring about tolerance cohort, UK (EAT)  Water hardness >255mg/L CaCO_3_  (Jabbar-Lopez, 2019)^37^ | N=1303 (317/986) | 75/141 | **P_INT_ = 0.008**  HR_INT_=2.72 (95% CI:2.03-3.66) | Report evidence of a gene-environment interaction between hard water and *FLG* loss-of-function mutations |
| VITAMIN D | R501X  2282del4  R2447X S3247X. | Cohort of acute bronchiolitis trial and general population of Norway  (Berents, 2016)^32^ | N=558 (103/455) | ?/41 | **P_INT_ > 0.13** | Report no evidence for an interaction between Vitamin D and *FLG* loss-of-function mutations |
| PTHALATE EXPOSURE – urine metabolites | P478S | CEAS  (Wang, 2015)^27^ | N=453 (106/347) | ?/? | P478S TT genotype interaction with MBP on AD (P for interaction=0.016) and P478S TT genotype interaction with MBzP on AD (P for interaction=0.049)  MBP levels <1.77  P478S  TT 0.54 (0.17–1.74)  TC 0.64 (0.23–1.81)  CC 1  MBP levels≧1.77  P478S  TT 4.74 (1.45–15.50)  TC 1.78 (0.53–5.99)  CC 1  MBzP levels <0.47  P478S  TT 0.87 (0.30–2.53)  TC 0.51 (0.18–1.49)  CC 1  MBzP levels≧0.47  P478S  TT 3.46 (1.03–11.58)  TC 2.15 (0.64–7.28)  CC 1 | The study suggests an interaction between *FLG* variants and phthalate exposure but also cites possible reverse causality.  Note also wide confidence intervals for association between high and low levels of MBP and MBzP |
| PHTHALATE EXPOSURE – household dust | 3321delA, Q1701X, S2554X, S2889X, S3296X, and K4022X | Hokkaido study on Environment and Children's Health  (Bamai, 2018) | N=296 (61/235) | 5/27 | DiNP **P_INT_<0.05**  *FLG*^-^ OR=0.27 (95% CI: 0.06-1.28)  *FLG* wild type OR=1.29 (95% CI: 0.98-1.69)  Total OR=1.17 (95% CI: 0.91-1.52)  Der1 **P_INT_<0.1**  *FLG*^-^ OR=0.60 (95% CI:0.23-1.55)  *FLG* wild type OR=1.10 (95% CI: 0.89-1.36) Total OR=1.04 (95% CI: 0.85-1.27) | The study suggests an interaction between DiNP and FLG loss-of-function mutations and very limited evidence of an interaction between Der1 and *FLG* genotype however interaction testing is based on N=5. |

*^^[[1]](#footnote-1)^^*

1. Abbreviations used in table:

   P_INT_ – P value for the interaction

   *FLG^-^* - Filaggrin genotype including one or more loss-of-function mutations

   HR – Hazard ratio

   OR – Odds ratio

   CI – Confidence interval

   RR1 – Risk ratio for *FLG* heterozygotes, each having one *FLG* loss-of-function mutation

   RR2 – Risk ratio for individuals who are homozygous compound heterozygous for *FLG* loss-of-function mutations

   ? – Data not available

   MEP - Monoethyl phthalate

   MBP - Monobutyl phthalate

   MBzP - Monobenzyl pthathlate

   5OHMEHP - Mono(2-ethyl-5-hydroxyhexyl)phthalate [↑](#footnote-ref-1)
