# Supplementary material for: What is the evidence for interactions between filaggrin null mutations and environmental exposures in the aetiology of atopic dermatitis? A systematic review
Source: Br J Dermatol. 2020 Feb 11;183(3):443–51. doi: 10.1111/bjd.18778 (PMC7496176; doi:10.1111/bjd.18778)
Supplement: Supplementary file 2 — Powerpoint S1 Journal Club Slide Set. [file BJD-183-443-s002.pptx]

## Slide 1
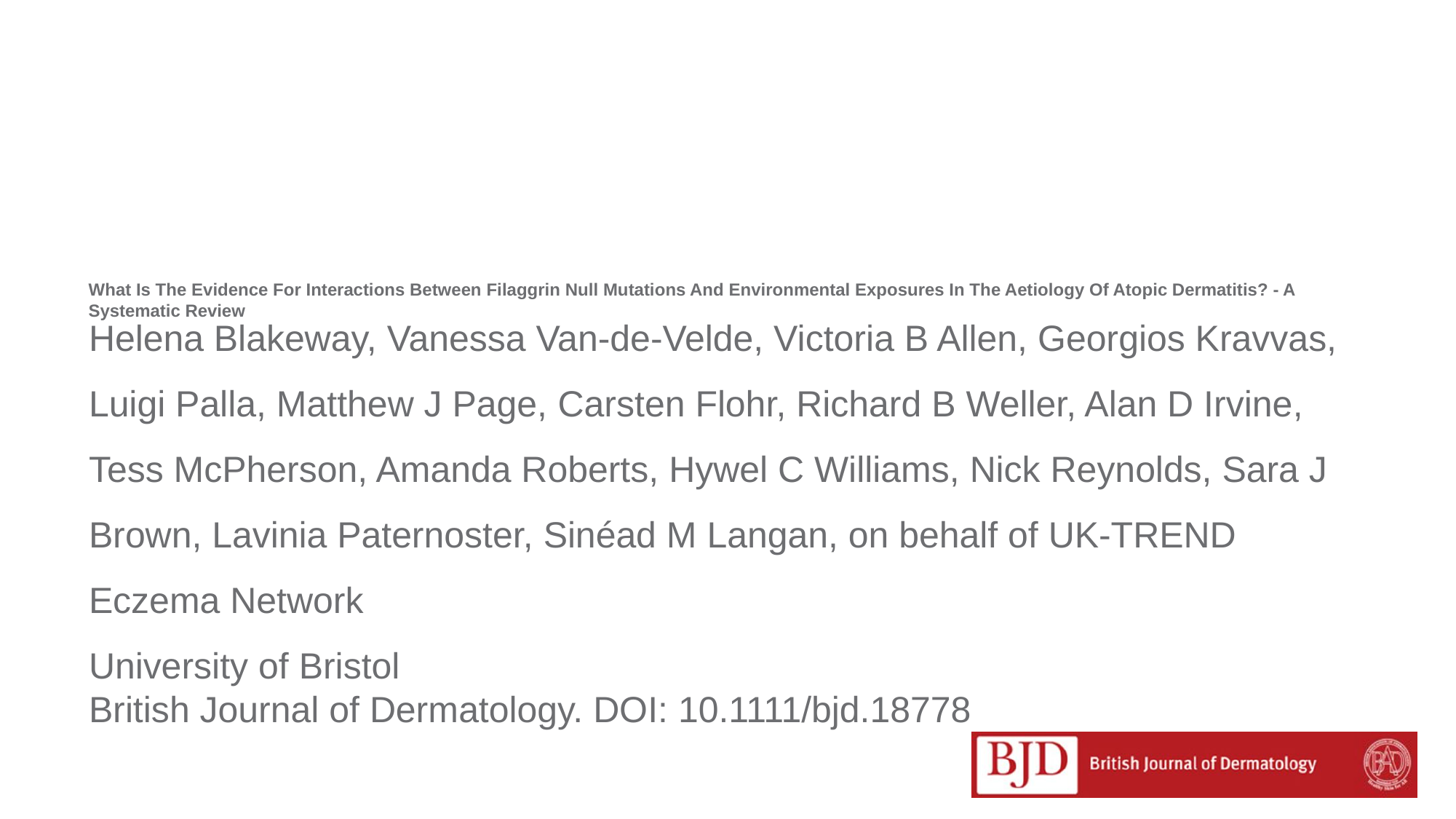

# What Is The Evidence For Interactions Between Filaggrin Null Mutations And Environmental Exposures In The Aetiology Of Atopic Dermatitis? - A Systematic Review
Helena Blakeway, Vanessa Van-de-Velde, Victoria B Allen, Georgios Kravvas, Luigi Palla, Matthew J Page, Carsten Flohr, Richard B Weller, Alan D Irvine, Tess McPherson, Amanda Roberts, Hywel C Williams, Nick Reynolds, Sara J Brown, Lavinia Paternoster, Sinéad M Langan, on behalf of UK-TREND Eczema Network
University of Bristol
British Journal of Dermatology. DOI: 10.1111/bjd.18778

## Slide 2
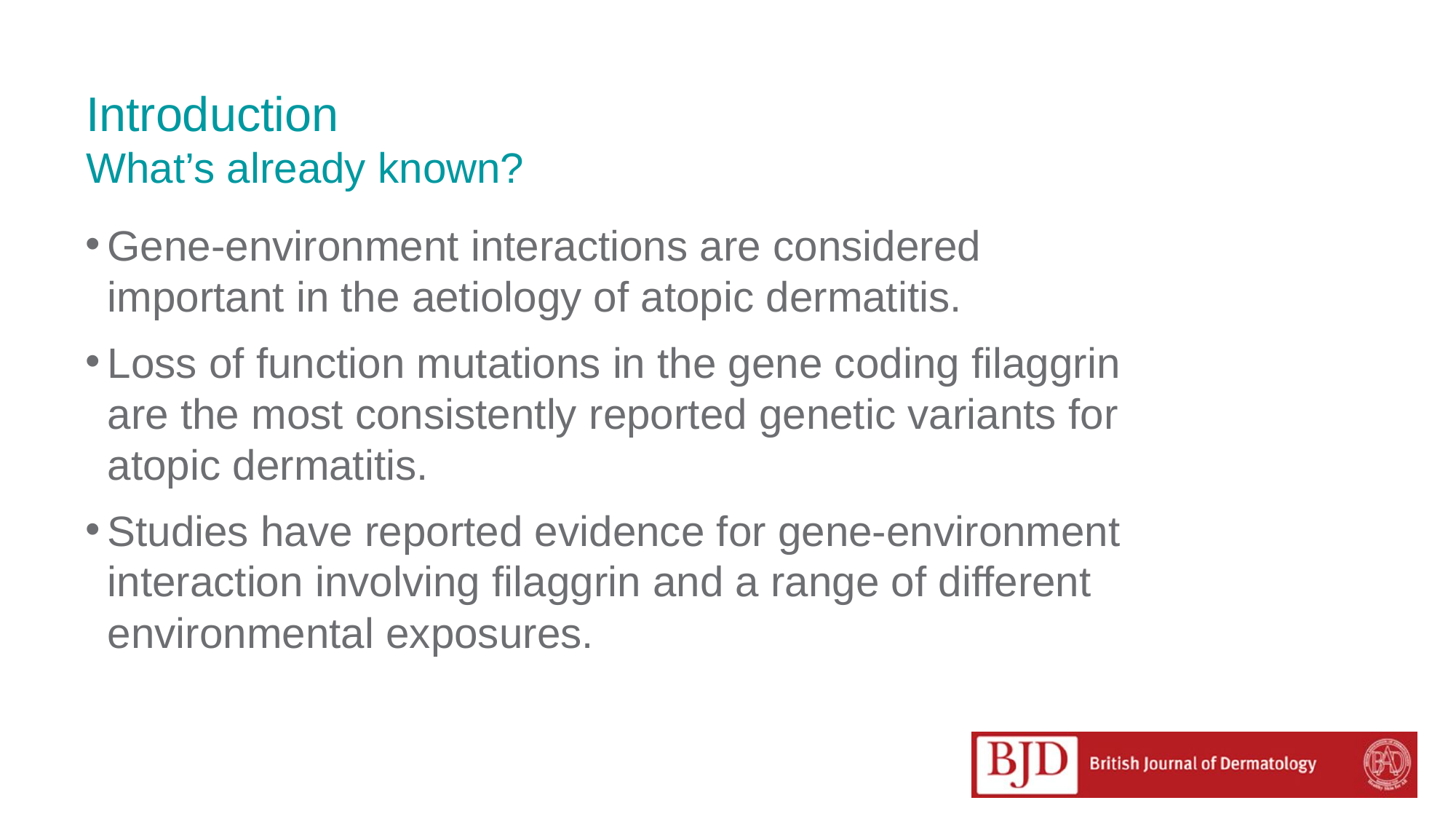

# Introduction What’s already known?
Gene-environment interactions are considered important in the aetiology of atopic dermatitis.
Loss of function mutations in the gene coding filaggrin are the most consistently reported genetic variants for atopic dermatitis.
Studies have reported evidence for gene-environment interaction involving filaggrin and a range of different environmental exposures.

## Slide 3
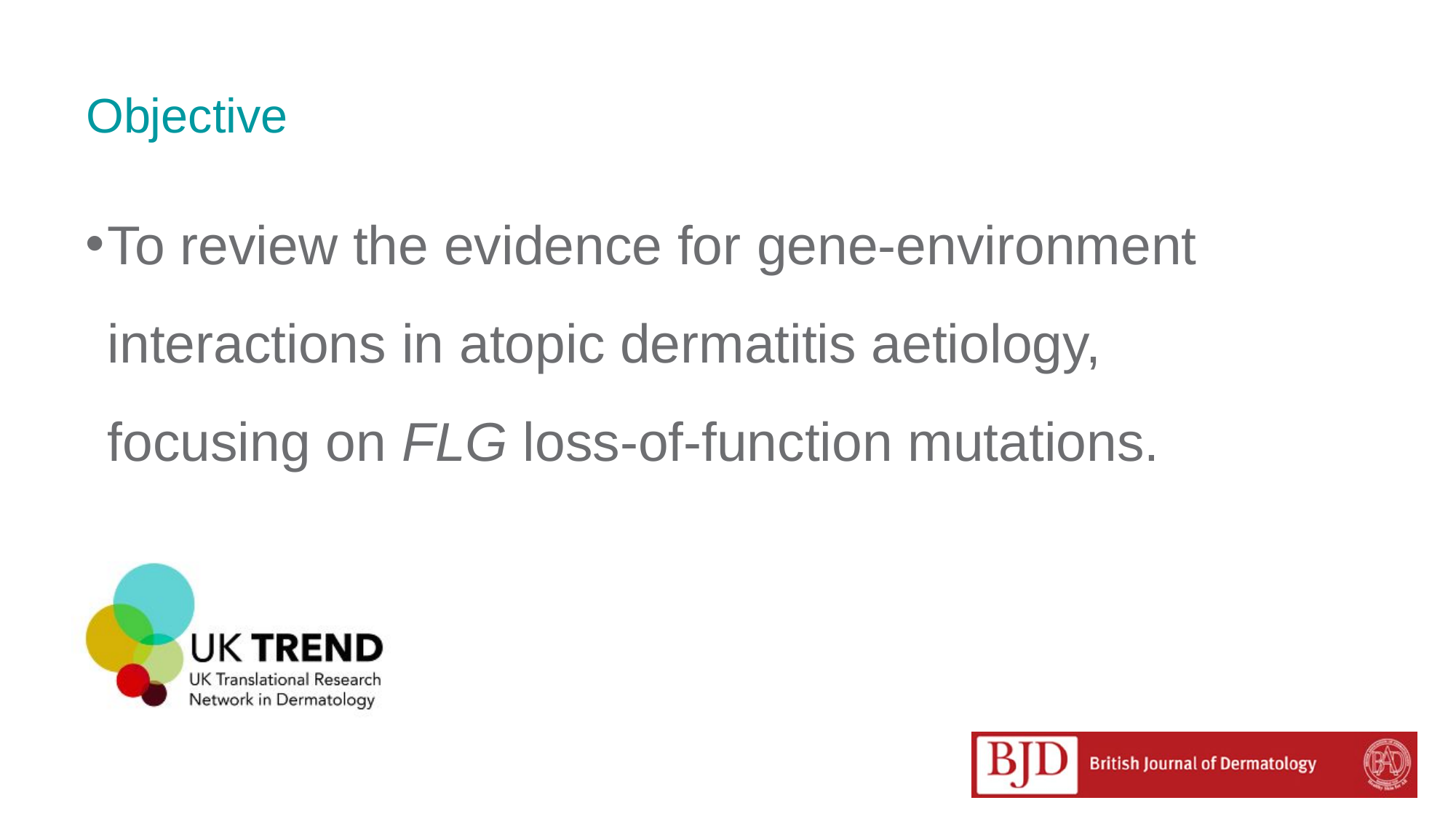

# Objective
To review the evidence for gene-environment interactions in atopic dermatitis aetiology, focusing on FLG loss-of-function mutations.

## Slide 4
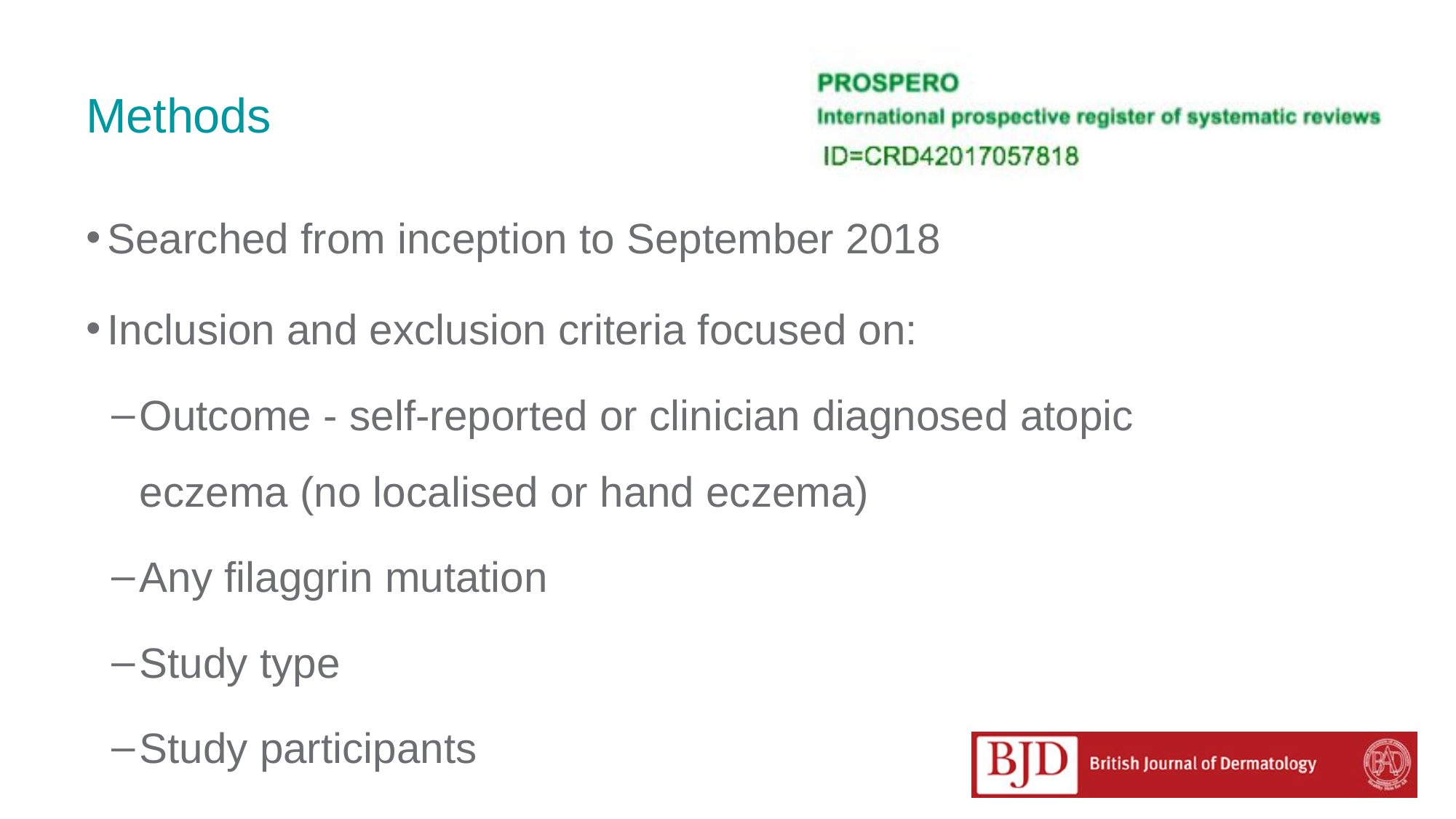

# Methods
Searched from inception to September 2018
Inclusion and exclusion criteria focused on:
Outcome - self-reported or clinician diagnosed atopic eczema (no localised or hand eczema)
Any filaggrin mutation
Study type
Study participants

## Slide 5
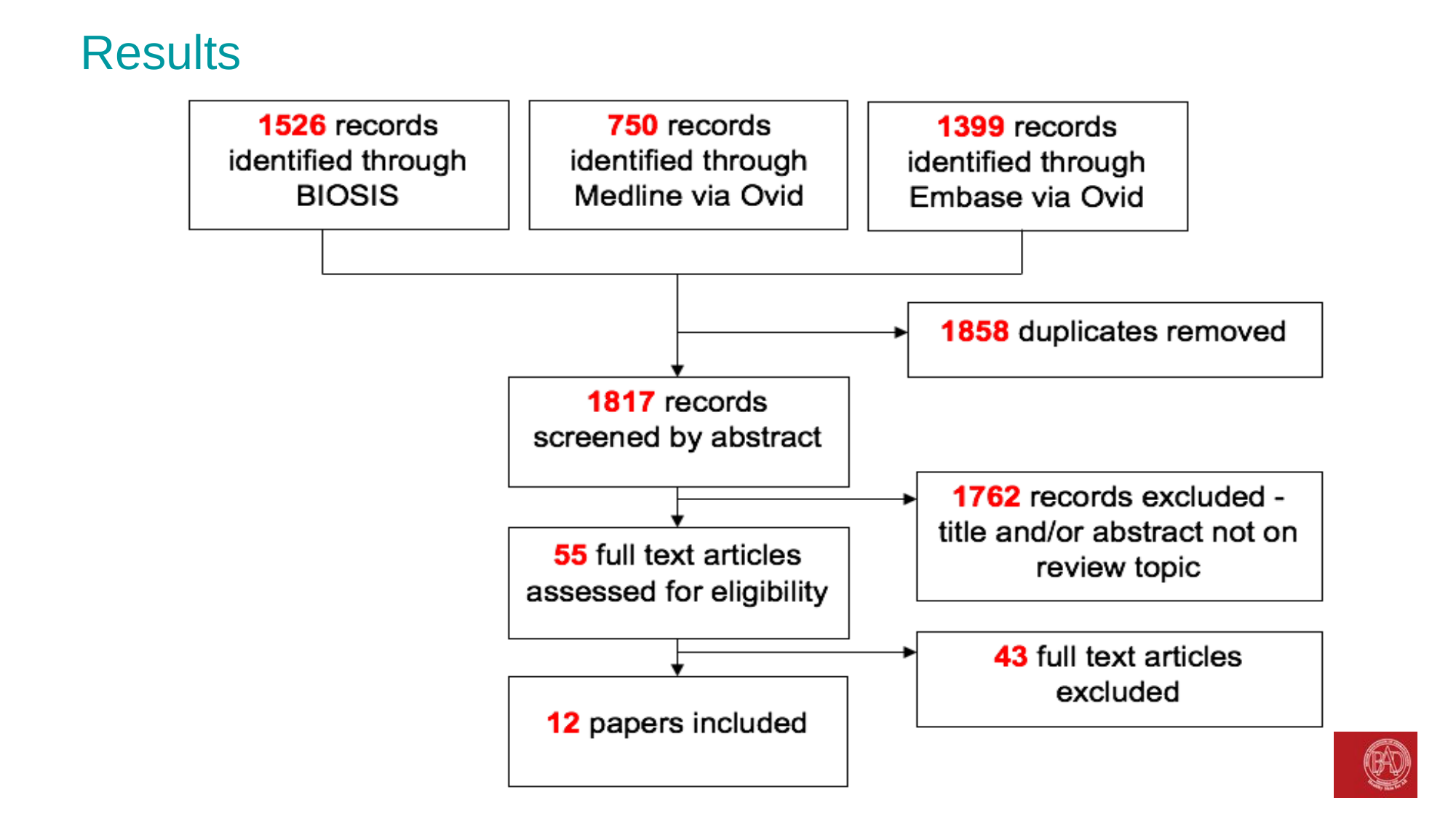

# Results

## Slide 6
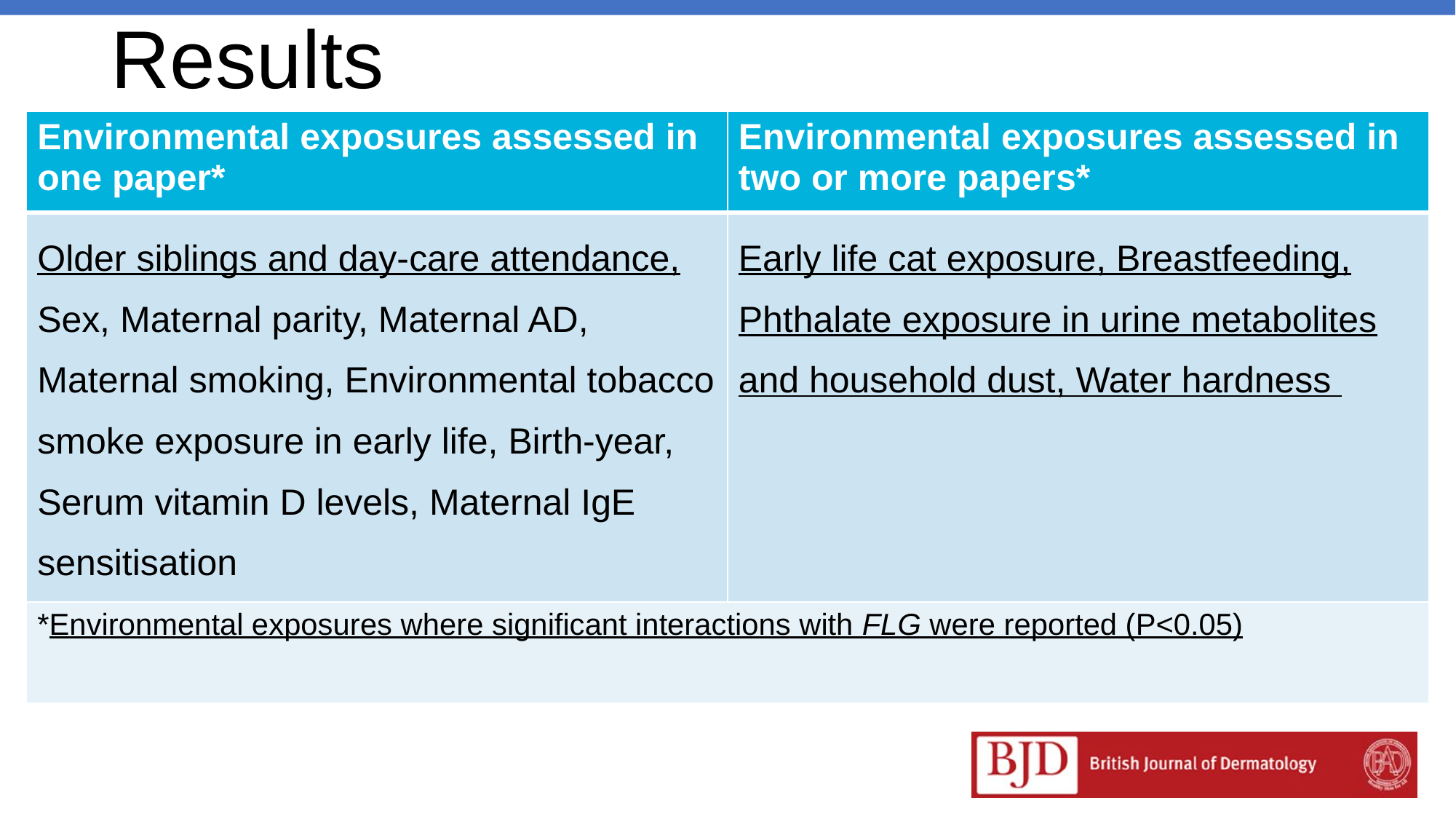

# Results
| Environmental exposures assessed in one paper\* | Environmental exposures assessed in two or more papers\* |
| --- | --- |
| Older siblings and day-care attendance, Sex, Maternal parity, Maternal AD, Maternal smoking, Environmental tobacco smoke exposure in early life, Birth-year, Serum vitamin D levels, Maternal IgE sensitisation | Early life cat exposure, Breastfeeding, Phthalate exposure in urine metabolites and household dust, Water hardness |
| \*Environmental exposures where significant interactions with FLG were reported (P<0.05) | |

## Slide 7
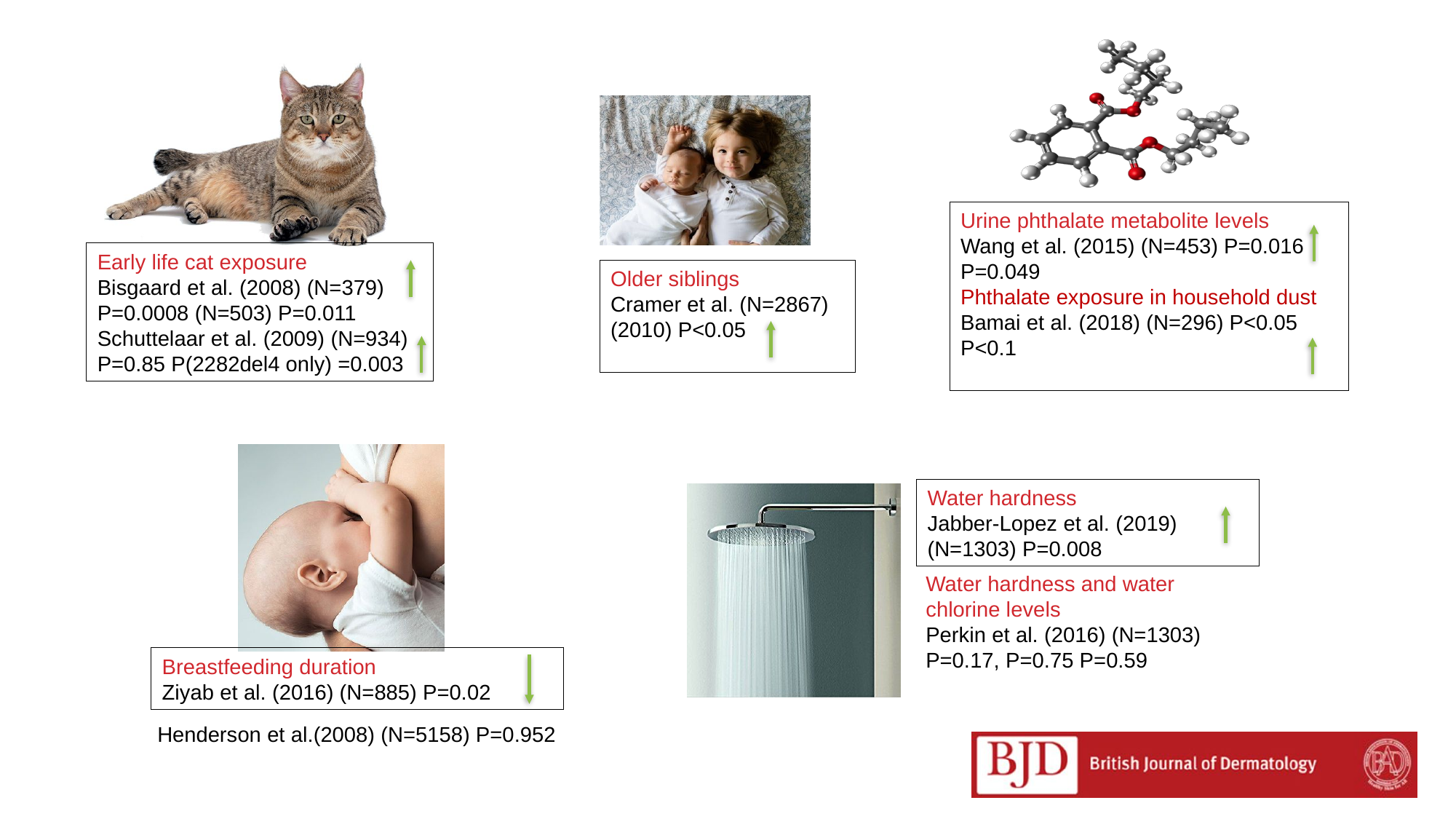

Urine phthalate metabolite levels
Wang et al. (2015) (N=453) P=0.016 P=0.049
Phthalate exposure in household dust
Bamai et al. (2018) (N=296) P<0.05 P<0.1
Early life cat exposure
Bisgaard et al. (2008) (N=379) P=0.0008 (N=503) P=0.011 Schuttelaar et al. (2009) (N=934) P=0.85 P(2282del4 only) =0.003
Older siblings
Cramer et al. (N=2867) (2010) P<0.05
Breastfeeding duration
Ziyab et al. (2016) (N=885) P=0.02
Henderson et al.(2008) (N=5158) P=0.952
Water hardness
Jabber-Lopez et al. (2019) (N=1303) P=0.008
Water hardness and water chlorine levels
Perkin et al. (2016) (N=1303) P=0.17, P=0.75 P=0.59

## Slide 8
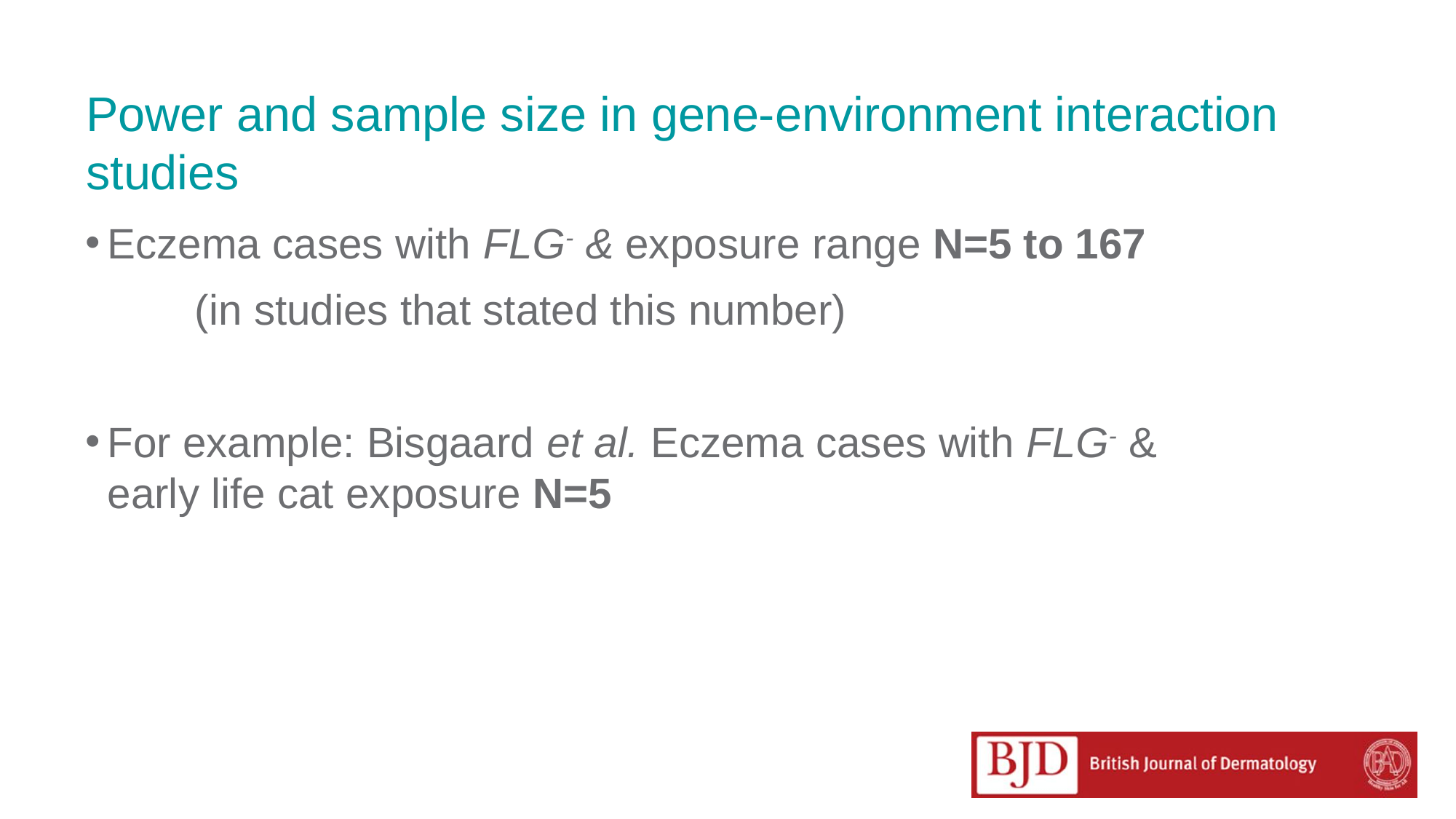

# Power and sample size in gene-environment interaction studies
Eczema cases with FLG- & exposure range N=5 to 167
	(in studies that stated this number)
For example: Bisgaard et al. Eczema cases with FLG- & early life cat exposure N=5

## Slide 9
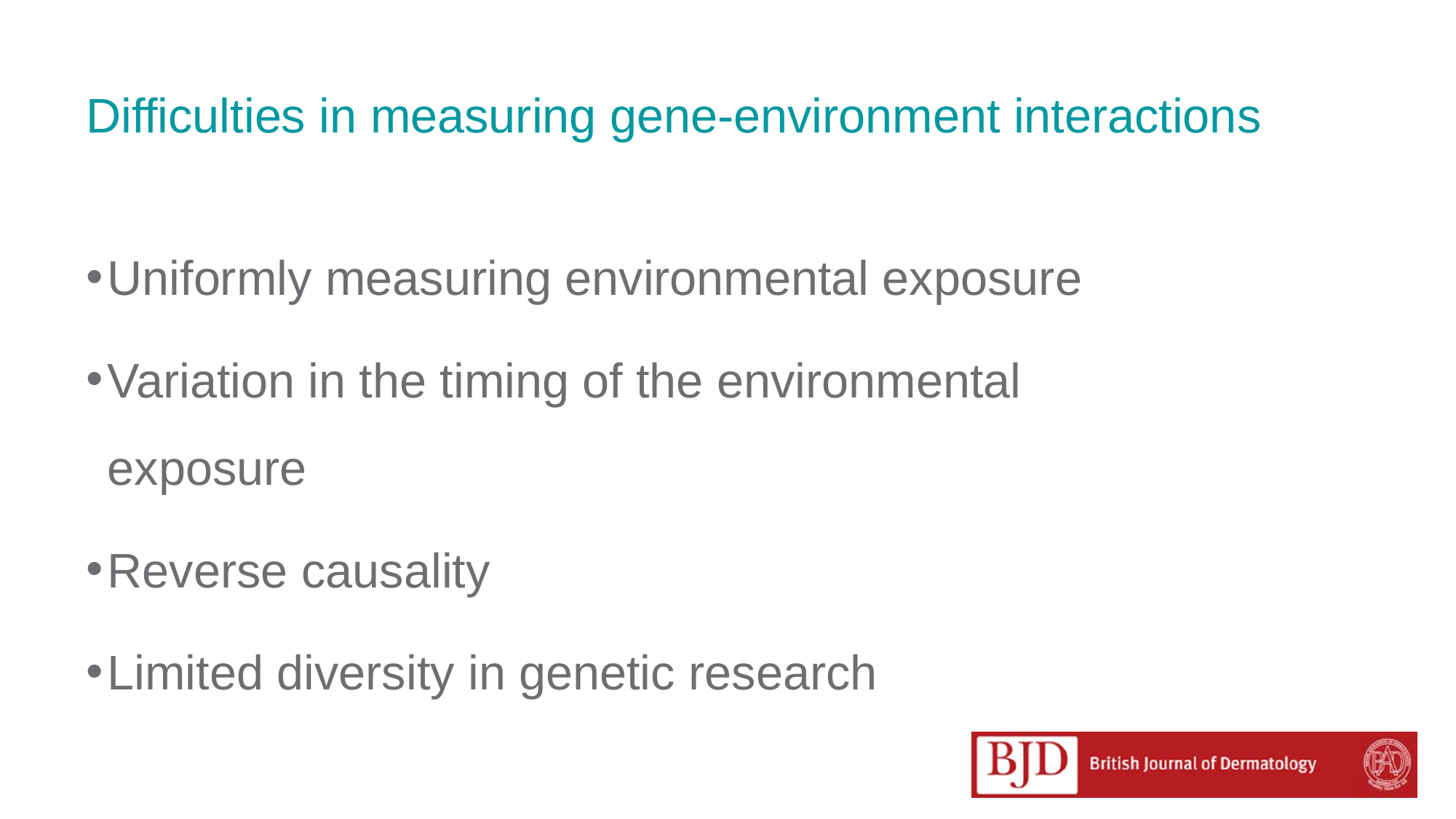

# Difficulties in measuring gene-environment interactions
Uniformly measuring environmental exposure
Variation in the timing of the environmental exposure
Reverse causality
Limited diversity in genetic research

## Slide 10
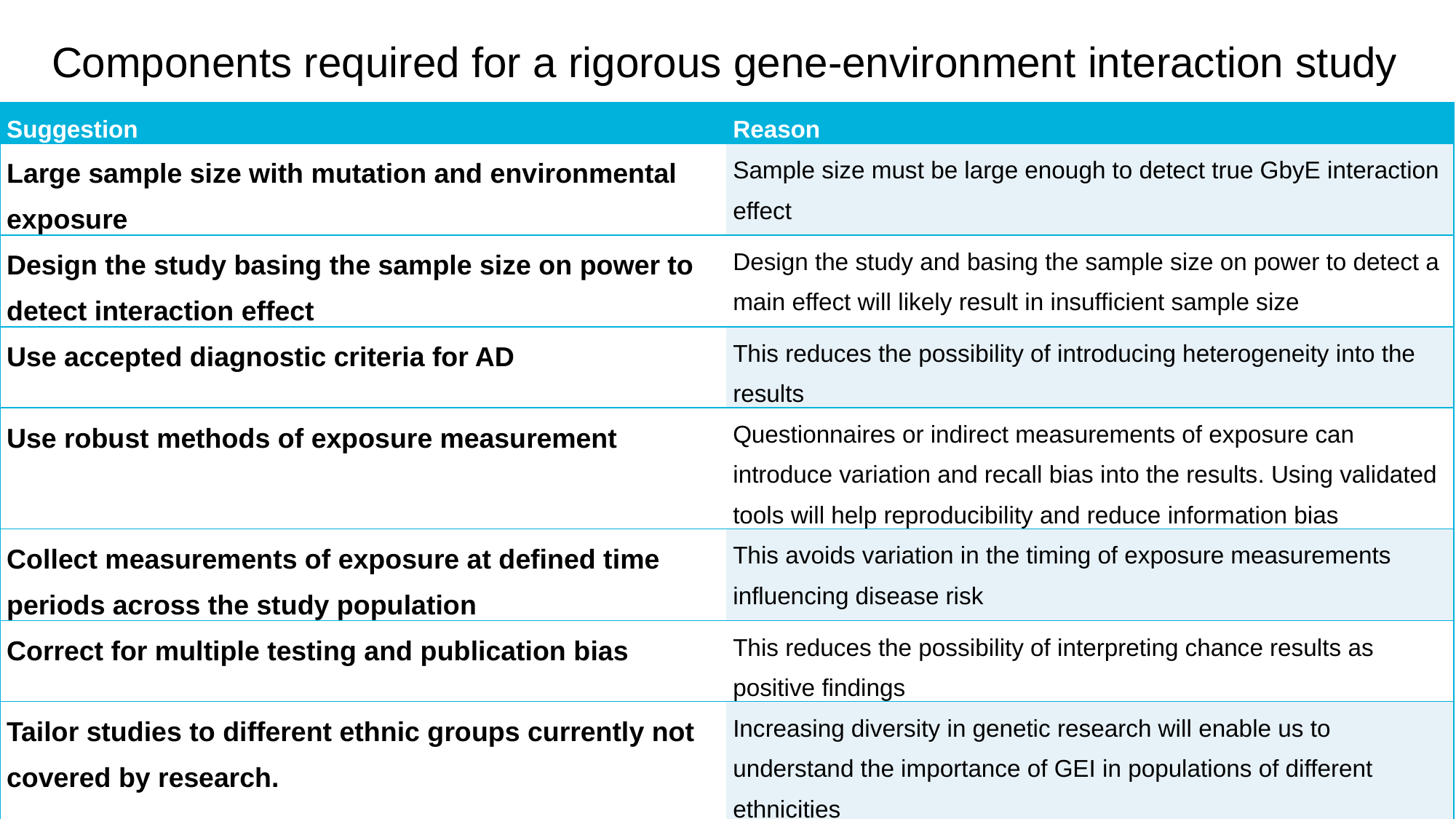

# Components required for a rigorous gene-environment interaction study
| Suggestion | Reason |
| --- | --- |
| Large sample size with mutation and environmental exposure | Sample size must be large enough to detect true GbyE interaction effect |
| Design the study basing the sample size on power to detect interaction effect | Design the study and basing the sample size on power to detect a main effect will likely result in insufficient sample size |
| Use accepted diagnostic criteria for AD | This reduces the possibility of introducing heterogeneity into the results |
| Use robust methods of exposure measurement | Questionnaires or indirect measurements of exposure can introduce variation and recall bias into the results. Using validated tools will help reproducibility and reduce information bias |
| Collect measurements of exposure at defined time periods across the study population | This avoids variation in the timing of exposure measurements influencing disease risk |
| Correct for multiple testing and publication bias | This reduces the possibility of interpreting chance results as positive findings |
| Tailor studies to different ethnic groups currently not covered by research. | Increasing diversity in genetic research will enable us to understand the importance of GEI in populations of different ethnicities |

## Slide 11
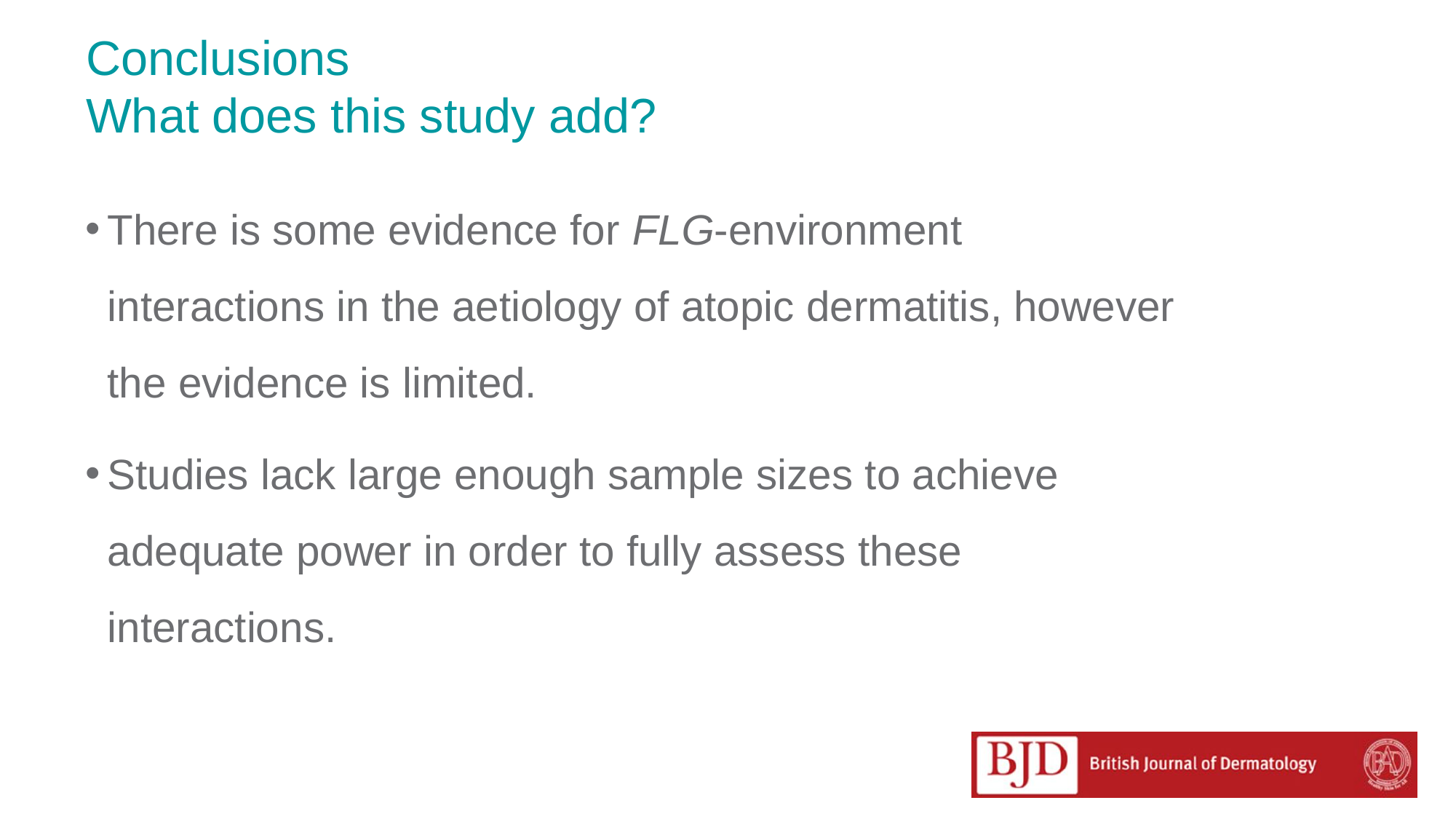

# Conclusions What does this study add?
There is some evidence for FLG-environment interactions in the aetiology of atopic dermatitis, however the evidence is limited.
Studies lack large enough sample sizes to achieve adequate power in order to fully assess these interactions.

## Slide 12
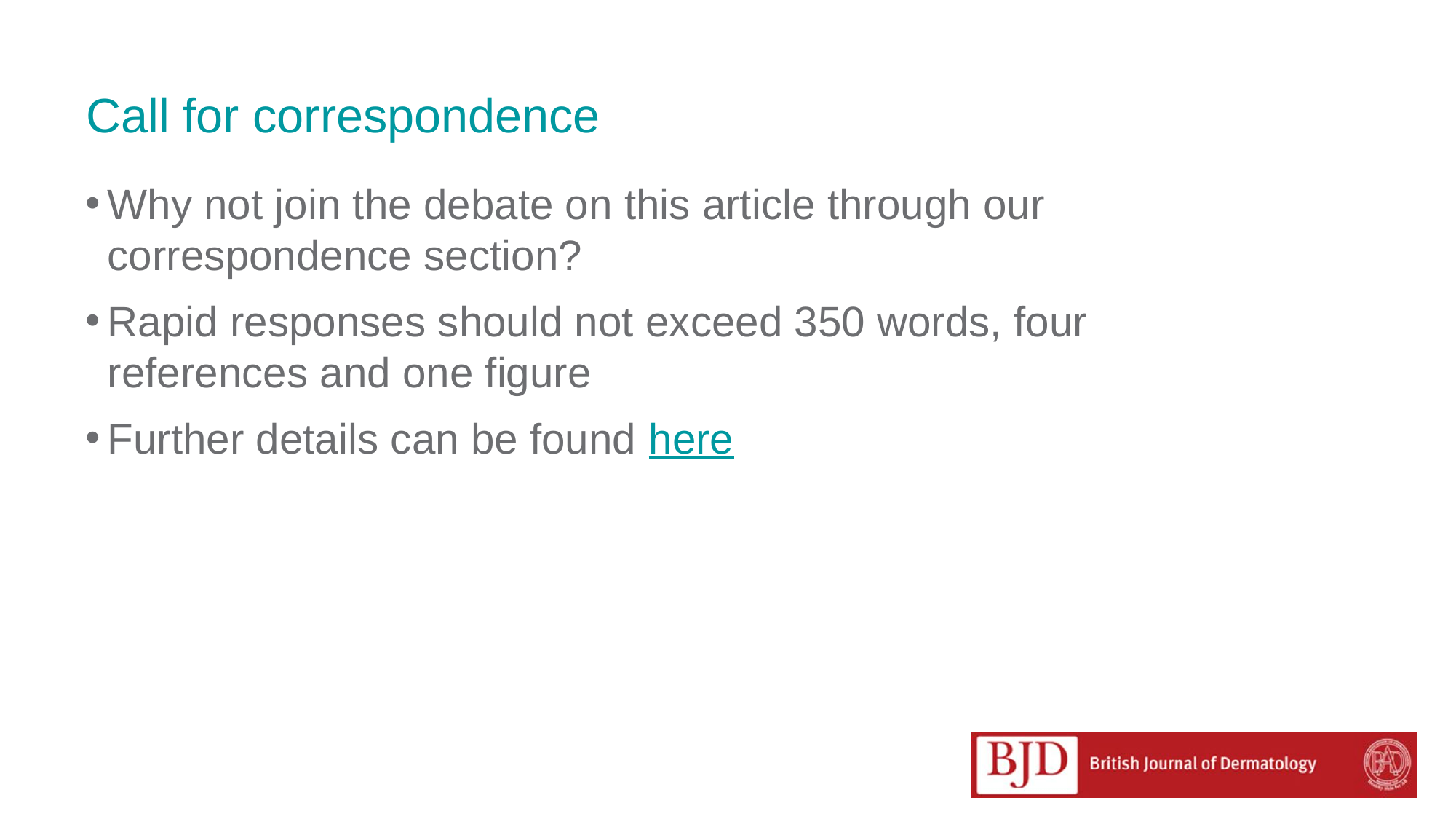

# Call for correspondence
Why not join the debate on this article through our correspondence section?
Rapid responses should not exceed 350 words, four references and one figure
Further details can be found here
